# Structural and functional characterization of the catalytic domain of a cell-wall anchored bacterial lytic polysaccharide monooxygenase from *Streptomyces coelicolor*

**DOI:** 10.1038/s41598-023-32263-7

**Published:** 2023-04-01

**Authors:** Amanda K. Votvik, Åsmund K. Røhr, Bastien Bissaro, Anton A. Stepnov, Morten Sørlie, Vincent G. H. Eijsink, Zarah Forsberg

**Affiliations:** 1 Faculty of Chemistry, Biotechnology, and Food Science, The Norwegian University of Life Sciences (NMBU), 1432 Ås, Norway; 2 INRAE, Aix Marseille University, UMR1163 Biodiversité et Biotechnologie Fongiques, 13009 Marseille, France

**Keywords:** Biochemistry, Enzymes, Structural biology, X-ray crystallography

## Abstract

Bacterial lytic polysaccharide monooxygenases (LPMOs) are known to oxidize the most abundant and recalcitrant polymers in Nature, namely cellulose and chitin. The genome of the model actinomycete *Streptomyces coelicolor* A3(2) encodes seven putative LPMOs, of which, upon phylogenetic analysis, four group with typical chitin-oxidizing LPMOs, two with typical cellulose-active LPMOs, and one which stands out by being part of a subclade of non-characterized enzymes. The latter enzyme, called *Sc*LPMO10D, and most of the enzymes found in this subclade are unique, not only because of variation in the catalytic domain, but also as their C-terminus contains a cell wall sorting signal (CWSS), which flags the LPMO for covalent anchoring to the cell wall. Here, we have produced a truncated version of *Sc*LPMO10D without the CWSS and determined its crystal structure, EPR spectrum, and various functional properties. While showing several structural and functional features typical for bacterial cellulose active LPMOs, *Sc*LPMO10D is only active on chitin. Comparison with two known chitin-oxidizing LPMOs of different taxa revealed interesting functional differences related to copper reactivity. This study contributes to our understanding of the biological roles of LPMOs and provides a foundation for structural and functional comparison of phylogenetically distant LPMOs with similar substrate specificities.

## Introduction

*Streptomyces*, the dominating genus of the actinobacteria phylum, are Gram-positive and mostly facultative aerobic, soil bacteria that possess a vast number of genes encoding Carbohydrate-Active enZymes (CAZymes), providing a multifaceted repertoire of enzymes for deconstruction of complex structural carbohydrates of high recalcitrance. Such carbohydrates include plant cell-wall polysaccharides such as cellulose and xylan, as well as chitin, the latter which is found in the cell walls of fungi and the exoskeletons of arthropod species (i.e., crustaceans and insects). The genome of the model representative *Streptomyces coelicolor* A3(2) has more than 240 CAZyme-encoding genes^1^, including glycoside hydrolases (GHs) from families 3, 5, 6, 9, 12, and 48 (i.e., typical cellulases^2^), in addition to GHs from family 18–20 (i.e., typical chitinases). Furthermore, *S. coelicolor* exhibits seven genes encoding lytic polysaccharide monooxygenases (LPMOs) that are all members of the auxiliary activity (AA) family 10 (AA10)^3,4^.

The AA class was relatively recently added to the CAZy database and holds redox-active enzymes that assist CAZymes in the degradation of biomass^5^. Currently, eight out of seventeen AA families contain LPMOs, viz*.* families AA9-11 and AA13-17^6–14^. LPMOs are widespread in Nature and are best known for their synergistic role with glycoside hydrolases in the conversion of cellulose and chitin^4,6–8,15,16^. While GHs use a hydrolytic mechanism to cleave glycosidic bonds of polysaccharides, LPMOs use a single copper co-factor, that upon reduction can activate H_2_O_2_^17,18^, and possibly also O_2_^6,19^ to generate a highly reactive oxygen species^17,18,20–22^ that is needed to oxidize either the C1 or the C4-carbon in the scissile glycosidic bond. Of note, the peroxygenase reaction (with H_2_O_2_) is orders of magnitude faster than the “monooxygenase” reaction (with O_2_)^18,23–25^. LPMO action makes the polysaccharide substrate more susceptible to the action of GHs, thus increasing the overall efficiency of the polysaccharide degradation process.

**Figure 1:**
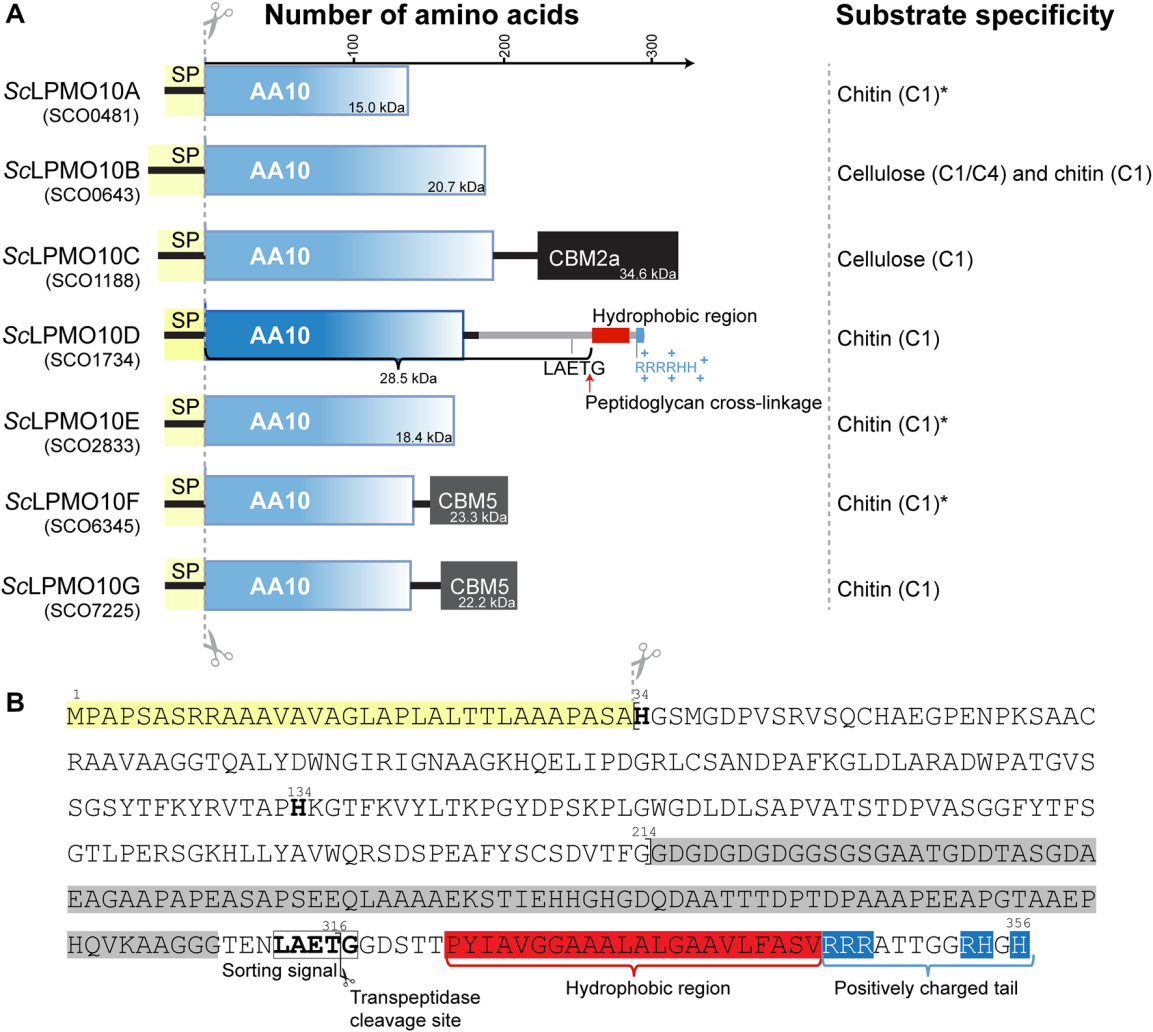
Overview of predicted *S. coelicolor* A3(2) LPMOs (**A**) and primary structure of *Sc*LPMO10D (**B**). (**A**) Shows the LPMO nomenclature that is based on the gene number shown below each enzyme in brackets, followed by domain architecture, the relative sizes (in number of amino acids and kDa) of the mature proteins and the substrates of the seven *S. coelicolor* LPMOs. The indicated substrates and oxidative regioselectivities of *Sc*LPMO10B^26^, *Sc*LPMO10C^8^, *Sc*LPMO10G^30^ and *Sc*LPMO10D (this study) are derived from experimental data, whereas the activity of the other LPMOs, marked with asterisks, is predicted based on sequence similarity and phylogenetic clustering (Fig. 2). (**B**) Shows the primary structure of *Sc*LPMO10D, including its signal peptide (yellow). The copper-coordinating histidines (His34 and His134) and the LAETG sorting signal are shown with bold letters. The region of low complexity (approximately residues 215–312), including a potential linker rich in Gly, Asp, Ala, and Ser, that connects the LPMO domain to the LAETG-motif transpeptidase (sortase) recognition site is shown on a grey background. The C-terminal hydrophobic region is shown on a red background and is followed by a C-terminal tail containing six positively charged residues (Arg and His) shown on a blue background. The mature *Sc*LPMO10D protein starts with His34 and ends with Thr316 crosslinked to the peptidoglycan of the *S. coelicolor* cell wall envelope. In this study a truncated version containing the catalytic domain (CD) only was used (*Sc*LPMO10D^CD^; residues 34-214).

**Figure 2:**
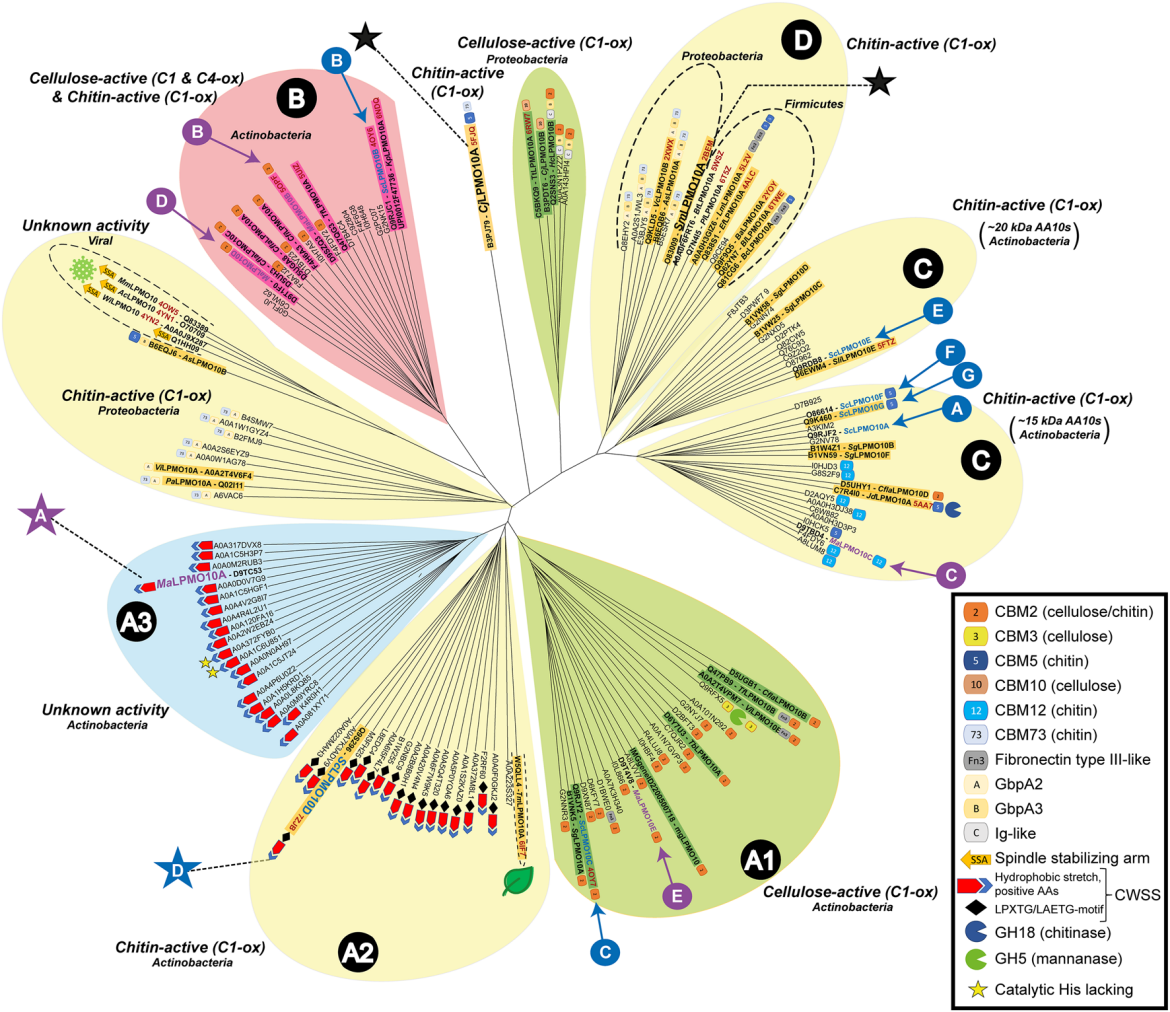
Phylogenetic tree built from 150 AA10 sequences. Highlighted enzymes have been experimentally characterized and are known to be strict C1 cellulose-oxidizers (green), show mixed C1/C4-oxidizing activity on cellulose and C1-oxidizing activity on chitin (pink) or known to oxidize the C1-carbon in chitin (yellow). Blue and purple labels indicate enzymes from *S. coelicolor* and *M. aurantiaca,* respectively*. Ma*LPMO10A, *Sc*LPMO10D, *Cj*LPMO10A and *Sm*LPMO10A, the four enzymes used in this study, are labelled by stars. The tree was built from catalytic domains only, but C-terminal domains are shown for each enzyme to highlight variation in domain architecture. The different domains found in the analyzed sequences include CBMs from families 2, 3, 5, 10, 12 and 73, fibronectin type III-like (Fn3) domains, immunoglobulin-like domains (Ig-like), GbpA2/GbpA3 domains, which have unknown functions and appear in GbpA-like LPMOs^39^, cell wall sorting signals (CWSS) and glycoside hydrolases belonging to families 5 (cellulases/mannanases) and 18 (chitinases). The yellow stars in subclade A3 indicate two sequences that lack one or two of the catalytic histidines, which are replaced by glutamines (see Supplemental Fig. S3). All sequences are of bacterial origin with the exception of four viral (cluster with chitin-active enzymes from Proteobacteria) and one of plant origin (subclade A2).

Fungal LPMOs, occupying families AA9, 11, 13, 14, and 16 have activity towards a range of polysaccharides including cellulose, cello-oligomers, various hemicelluloses, chitin, and starch^7,9,10,13,14^. On the other hand, for bacterial LPMOs, which dominate the AA10 family, only activity towards cellulose or chitin, or both has been described^6,8,26^. In addition, LPMO genes tend to be abundant in fungal genomes, some with numbers reaching >30^27^, whereas most bacterial genomes only have one or two LPMO genes, with the notable exception of some members of the *Streptomyces* genus^28^. Two of the seven LPMOs in the genome of *S. coelicolor* A3(2) (Fig. 1), *Sc*LPMO10B and *Sc*LPMO10C (previously known as CelS2), have been extensively studied and shown to target cellulosic substrates^8,17,26,29^. Recent transcriptomic studies revealed that *Sc*LPMO10E and *Sc*LPMO10G were significantly upregulated when *S. coelicolor* was grown on chitin as a sole carbon source, and recombinant *Sc*LPMO10G was shown to possess chitin oxidizing activity^30^. Phylogenetic analysis showed that the latter two LPMOs (i.e., *Sc*LPMO10E and *Sc*LPMO10G) together with *Sc*LPMO10A and *Sc*LPMO10F cluster with known chitinolytic AA10s^31,32^, whereas *Sc*LPMO10D clusters in a novel subclade of uncharacterized enzymes (Fig. 2).

In this study, we describe the structural and functional characterization of *Sc*LPMO10D, which belongs to a subclade of hitherto uncharacterized enzymes. Of note, enzymes in this subclade are not only distinctive because of sequence variation in the catalytic domain but they are also special in that their sequences predict them to be cell-wall anchored (Fig. 1). We have compared the functional properties of the catalytic domain (CD) of *Sc*LPMO10D (called *Sc*LPMO10D^CD^), which turned out to be active on chitin, to the properties of two well-characterized chitin-oxidizing LPMOs (*Sm*LPMO10A and *Cj*LPMO10A^CD^) that appear in distinct subclades of chitin-active LPMOs in the AA10 phylogenetic tree (Fig. 2). In addition to revealing functional properties of *Sc*LPMO10D, the results show that the three chitin-active enzymes differ considerably in terms of substrate-binding, redox potential, H_2_O_2_ production, stability under turnover conditions and the EPR spectra of their catalytic copper sites.

## Results

### *Sc*LPMO10D belongs to an uncharacterized AA10 clade populated by cell wall-anchored LPMOs

In 2008 Walter and Schrempf described a surface-located carbohydrate-binding protein, belonging to the carbohydrate-binding module (CBM) family 33, from *S. coelicolor* A3(2) called CbpC^33^. Today the proteins and domains formerly referred to as CBM33 are known as AA10-type LPMOs or LPMO10s^3,6^. In the study by Walter and Schrempf, it was shown that expression of CbpC was induced when chitin, cellulose or cellobiose, but not glucose, were used as the sole carbon source. It was also shown that the protein bound strongly to Avicel, and, to a lesser extent to chitinous substrates. Unlike other AA10-type LPMOs, CbpC possesses a C-terminal cell wall sorting signal (CWSS) and by using polyclonal anti-CbpC antibodies Walter and Schrempf showed that the protein was bound to the bacterial cell wall whereas a truncated version of CbpC, lacking the CWSS was translocated to the supernatant^33^.

CbpC, hereafter called *Sc*LPMO10D (Uniprot ID: Q9S296; Fig. 1), consists of 356 amino acids (Fig. 1B), making up an N-terminal signal peptide (residues 1–33), a family AA10 LPMO domain (residues 34–214; *Sc*LPMO10D^CD^), a low complexity region including a potential linker rich in glycine, alanine, aspartic acid, and serine (approximately residues 215–312), and a C-terminal CWSS (residues 313–356). The CWSS includes a characteristic LAETG-motif, followed by a stretch of disordered hydrophobic amino acids and a C-terminal tail rich in positively charged residues^34^. The LAETG-motif represents a *Streptomyces* specific sorting signal, substituting the LPXTG-motif found in the cell wall-anchored proteins of *Staphylococcus* and *Streptococcus* species^35,36^. The LAETG-motif makes up a recognition site for class E sortases (SrtE1), a type of transpeptidase that cleave and crosslink target proteins to the peptidoglycan matrix of the Gram-positive cell wall^37^.

Phylogenetic analysis of 150 AA10 LPMO sequences (catalytic domains only), including 45 enzymes that have been experimentally characterized (see Supplementary Table S1), showed that *Sc*LPMO10D^CD^ does not cluster with any of the established subclades of AA10 sequences (Fig. 2). In a previous phylogenetic study by Book et al.^31^ two clades were defined: Clade I comprises subclades C and D, which both harbor chitin-oxidizing LPMOs, while Clade II comprises subclades A and B, of which subclade B harbors enzymes with mixed C1- and C4-oxidizing cellulose-activity in addition to C1-oxidizing activity on chitin, and subclade A harbors C1-oxidizing cellulose-active enzymes and the cell wall-anchored AA10s that are in focus in this study. Subclade A groups into three distinct clusters (Fig. 2) and has therefore, in this study, been divided into subclades A1-A3, of which subclade A1 contains C1-oxidizing cellulose active enzymes^26^. Subclade A2 comprises the *Sc*LPMO10D-like enzymes with LAETG motifs which, as shown below, have C1-oxidizing chitin activity, whereas subclade A3 contain proteins with as yet unknown activities (see below). Interestingly, subclade A2 also contains the only AA10 found in plants, namely Tma12 from the fern *Tectaria macrodonta* that has been shown to have insecticide properties^38^, but no oxidative activity has been demonstrated to date. With Tma12 as an exception, subclade A exclusively harbors AA10s of actinobacterial origin, thus Tma12 may suggest an event of horizontal gene transfer from actinobacteria to plants.

In this study we have produced the catalytic domain of *Sc*LPMO10D (previous CbpC^33^) and made attempts to produce the catalytic domain of *Micromonospora aurantiaca* LPMO10A (*Ma*LPMO10A^CD^, see Fig. 2 and Supplementary Fig. S1), which are both found in subclades of uncharacterized AA10s i.e., A2 and A3, respectively. However, despite several attempts *Ma*LPMO10A^CD^ could not be produced as a soluble protein and its functional properties could thus not be studied.

### Crystal structure of the catalytic domain of *Sc*LPMO10D

**Figure 3:**
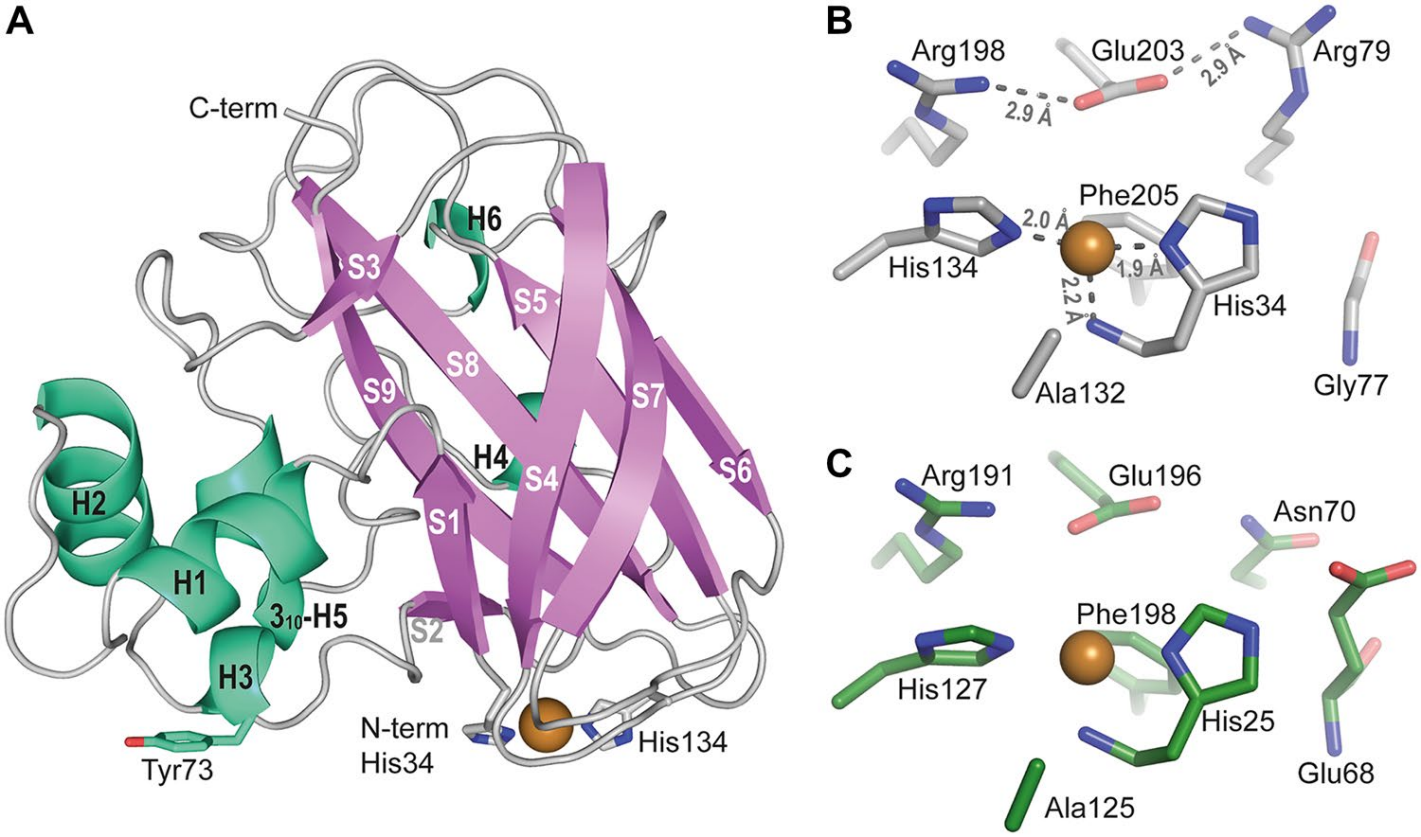
Three-dimensional structure of *Sc*LPMO10D^CD^. (**A**) Shows a cartoon representation of the catalytic domain, highlighting secondary structure elements (PDB 7ZJB). The six α-helices (including one 3_10_ helix; H5) are labelled H1-H6 and are colored cyan-green; strands are labelled S1-S9 and are colored violet. The copper atom is shown as an orange sphere and is coordinated by two histidines, N-terminal His34 and His134, shown with stick representation. Tyr73 is also shown with stick representation as it is known to be important for substrate binding in several characterized LPMO10s^45,46^. (**B**) Shows a close-up view of the catalytic center and displays the distances between the ligands and the copper cofactor in Ångström (Å). Conserved secondary sphere residues Ala132 and Phe205, which help in shaping the copper site, are also shown, as well as the unusual Arg198-Glu203-Arg79 arrangement. For comparison, (**C**) shows the catalytic center of the closest structurally characterized homologue of *Sc*LPMO10D, *Tm*LPMO10A (also known as Tma12), which is predicted to be chitin-active and lacks this Arg-Glu-Arg arrangement.

The structure of the catalytic domain of *Sc*LPMO10D (residues 34–214, lacking the linker and the CWSS), hereafter called *Sc*LPMO10D^CD^, was determined to 1.37 Å resolution with a single molecule in the asymmetric unit using the crystal structure of LPMO10A from *Tectaria macrodonta* (Tma12; PDB ID: 6IF7^38^) as the starting model (Table 1). The structure shows the typical LPMO fold with a central β-sandwich built up by two distorted β-sheets that are connected by several loops and helices (Fig. 3). The first β-sheet is formed by three antiparallel β-strands (S1, S4 and S7) and the second β-sheet is built up by four antiparallel strands (S5, S6, S8 and S9). Two small β-strands (S2 and S3) are found before and after the long “L2 loop” that holds most of the α-helices (H1, H2, H3 and H5), in which one is a 3_10_-helix (H5). Of note, most of the structural diversity observed among AA10s confines to the L2 loop, which accounts for approximately half of the substrate-binding surface^40,41^.

**Table 1:** Crystal data, diffraction data and refinement statistics for the *Sc*LPMO10D^CD^ structure (PDB code 7ZJB).

Crystal data	
Space group	P 4_3_2_1_2
Crystal parameters	a = 59.08 Å, b = 59.08 Å, c = 145.72 Å
	α = 90°, β = 90°, γ = 90°

Data collection	
X-ray source	ESRF, ID23-1
Resolution (Å)^a^	54.81–1.37 (1.42–1.37)
Wavelength (Å)	0.97702
Temperature (K)	100
Number of unique reflections	55,188 (5301)
Completeness^a^	99.8 (99.6)
Redundancy^a^	12.6 (12.7)
CC half^a^	1.0 (0.789)
I/s(I)^a^	22.5 (1.6)
R_merge_^b^	0.050 (1.369)

Refinement statistics	
*R*_cryst_^c^	0.129
*R*_free_^d^	0.152
Wilson B-factor (Å^2^)	19.8
Ramachandran plot, in most favored/other allowed regions (%)	100/0
Added waters	214

^a^Values for outer shell in paranthesis.

^b^$${R}_{sym}= \sum |I- \langle I\rangle |/\sum I$$

^c^$${R}_{cryst}= \sum (|{F}_{obs}\left| - \left|{F}_{calc}\right|\right)/\sum |{F}_{obs}|$$

^d^${R}_{free}$ is the ${R}_{cryst}$ value calculated on the 5% reflections excluded for refinement.

The active site of *Sc*LPMO10D is formed by His34 and His134, which coordinate the bound copper cofactor in a T-shaped geometry (Fig. 3B). No water molecules were found adjacent to the copper, which indicates that the copper had been photo-reduced during data collection^42,43^. The residues in the secondary coordination sphere resemble those found in C1-oxidizing cellulose-active AA10 LPMOs, such as *Sc*LPMO10C^26^ and in the atypical chitin-active *Cj*LPMO10A^44^. The second sphere includes a glutamate (Glu203) in what has been called the ‘gate-keeping' position^18^ that has a close interaction with an arginine (Arg198). This glutamate is thought to affect polysaccharide binding, allow diffusion of small molecules (such as H_2_O, O_2_ or H_2_O_2_) through an active site access tunnel formed at the interface between the LPMO and the polysaccharide^45^ and, in the case of H_2_O_2_, activation of the co-substrate^18^. In *Sc*LPMO10D, an additional arginine (Arg79) is interacting with Glu203 that together with Arg198 forms a remarkable arrangement that has, to the best of our knowledge, not been observed in any other LPMO (Fig. 3B).

The closest structurally characterized homologue of *Sc*LPMO10D^CD^ is the fern LPMO *Tm*LPMO10A^38^ (Fig. 3C). The sequence identity between *Sc*LPMO10D^CD^ and *Tm*LPMO10A is 57.4%, whereas the sequence identity with well-studied chitin-active *Cj*LPMO10A^CD^ and cellulose-active *Sc*LPMO10C^CD^, is 42.7% and 41.5%, respectively. The sequence identity between *Sc*LPMO10D^CD^ and chitin-active *Sm*LPMO10A is only 30.6%. *Ma*LPMO10A, a putatively membrane bound AA10 LPMO lacking the LAETG/LPXTG-motif (see above and Supplementary Fig. S1), which could not be produced, shows a sequence identity of 44% to *Sc*LPMO10D^CD^.

**Figure 4:**
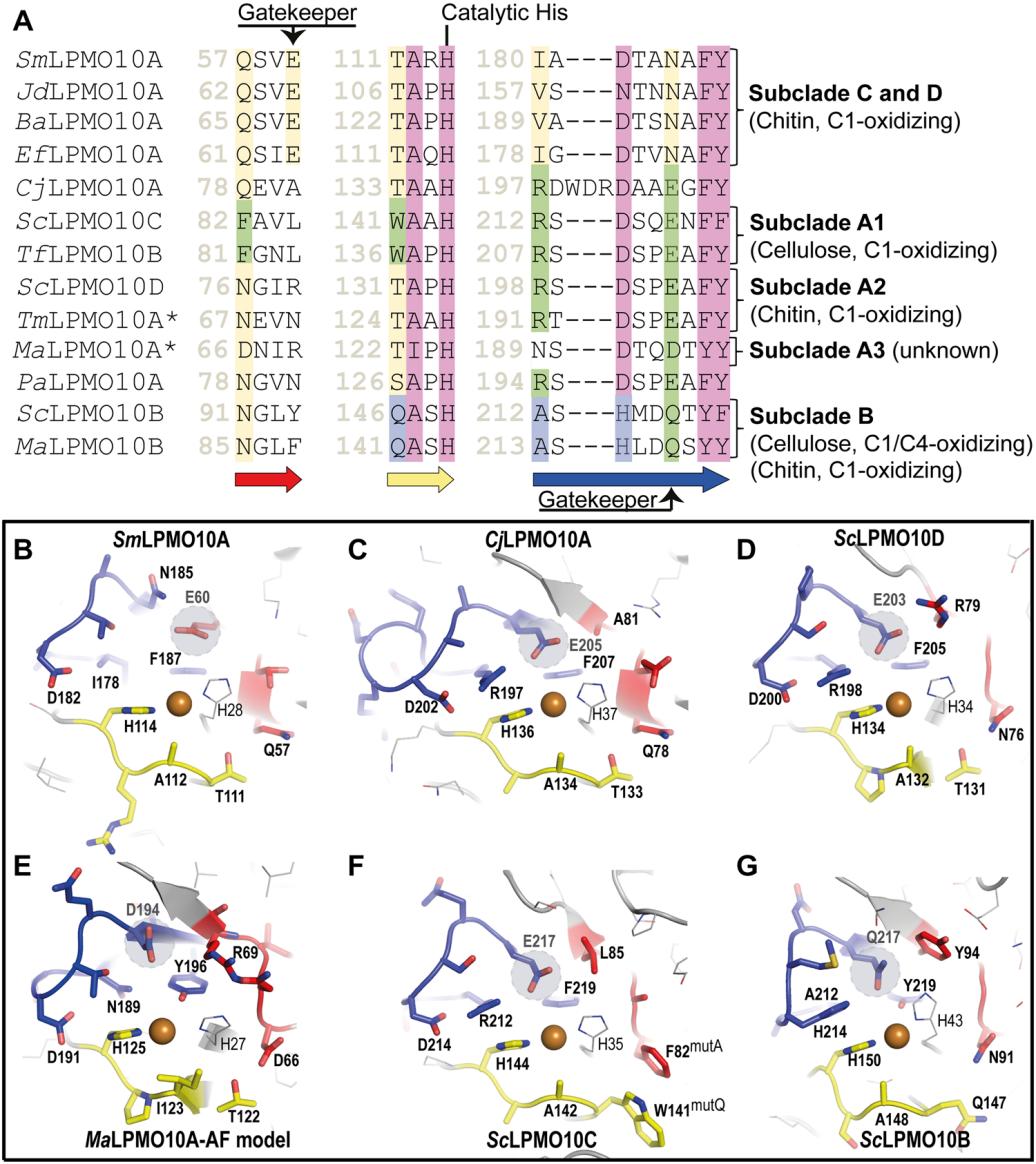
Structural comparison of active site residues in *Sc*LPMO10D and LPMOs from different subclades in the AA10 phylogenetic tree. (**A**) Show sections of a structural sequence alignment of 13 selected LPMO10s covering the subclades that were defined in the study by Book et al.^31^ and includes seven chitin-oxidizing LPMO10s, *Sm*LPMO10A (PDB ID: 2BEM; also known as CBP21), *Jd*LPMO10A (PDB ID: 5AA7), *Ba*LPMO10A (PDB ID: 2YOY), *Ef*LPMO10A (PDB ID: 4ALC), *Cj*LPMO10A (PDB ID: 5FJQ), *Sc*LPMO10D (PDB ID: 7ZJB) and *Pa*LPMO10A (also known as CbpD, PDB ID: 7SQX), and four cellulose-active LPMO10s, namely *Sc*LPMO10C (PDB ID: 4OY7; also known as CelS2), *Tf*LPMO10B (also known as E8; no crystal structure available), *Sc*LPMO10B (PDB ID: 4OY6) and *Ma*LPMO10B (PDB ID: 5OPF). The green- and yellow-colored amino acids indicate similarity to typical cellulose-active and typical chitin-active LPMOs, respectively. Blue colored amino acids are specific for subclade B LPMO10s, which have mixed regioselectivity and substrate specificity and whose active site resembles the typical active sites of fungal AA9 LPMOs. Purple residues indicate fully or highly conserved residues, including one of the catalytic histidines. Asterisks indicates that activity has not been reported. Note that the structurally conserved ‘gatekeeper’ residue, which is a Glu in LPMOs from Subclade C and D, has different positions in the LPMO sequence, depending on the subclade, as indicated above and under the alignment. The arrows under the alignment are labelled according to colors used in (**B**–**G**), showing the active sites of *Sm*LPMO10A (**B**), *Cj*LPMO10A (**C**), *Sc*LPMO10D (**D**), *Ma*LPMO10A (**E**), *Sc*LPMO10C (**F**) and *Sc*LPMO10B (**G**). Note that the structure of *Ma*LPMO10A was not determined experimentally but predicted using AlphaFold^48^.

Figure 4 shows sequence details and structures of the catalytic centers of these LPMOs. Generally, it is worth noting the considerable variation in second sphere residues, the functional implications of which are largely unknown. It is also worth noting that the arrangement of a catalytically crucial glutamate (Glu203) interacting closely with two positively charged residues (Arg79 and Arg198) really stands out from the active site arrangements found in other LPMOs (see also Fig. 3). Like *Cj*LPMO10A^44^, *Sc*LPMO10D shows a “hybrid”-type catalytic center with features known from both cellulose-active and chitin-active LPMO10s. While Arg198 and Glu203 in *Sc*LPMO10D are common to cellulose-active LPMO10s, Asn76 and Thr131 are common to chitin-active LPMOs (Fig. 4). Interestingly, a recent study in which a mutant library of cellulose-oxidizing *Sc*LPMO10C was screened for chitinolytic activity showed that both Phe82 (corresponding to Asn76 in *Sc*LPMO10D) and Trp141 (corresponding to Thr131) had to be mutated to obtain such activity^47^. A structural model (AlphaFold) of *Ma*LPMO10A shows an unusual active site that has not been seen in other LPMOs (Fig. 4E and Supplementary Fig. S2) where the residues corresponding to Arg198 and Glu203 in *Sc*LPMO10D are replaced by Asn189 and Asp194, respectively. Two residues that help shaping the copper site and that are commonly alanine and phenylalanine in AA10s (see Fig. 4), are replaced by an isoleucine and a tyrosine (Ile123 and Tyr196 in *Ma*LPMO10A), in which the latter residue is fully conserved in sequences belonging to subclades A3 and B. Of note, two of the sequences that cluster with *Ma*LPMO10A (labelled with yellow stars in Fig. 2) lack one or both copper-binding histidines (Supplementary Fig. S3).

### Substrate specificity of *Sc*LPMO10D^cd^

*Sc*LPMO10D^CD^ was tested on several known LPMO substrates including α- and β-chitin, chito-oligomers (DP5-6), phosphoric acid swollen cellulose (PASC), Avicel PH-101, bacterial microcrystalline cellulose (BMCC) and peptidoglycan isolated from *Streptomyces.* Reaction products were analyzed by MALDI-ToF MS, HPAEC-PAD and UHPLC—with only oxidized products being produced for reactions with α- and β-chitin (Supplementary Fig. S4). The degree of polymerization of the soluble products ranged from 4–7 and 4–9 for α- and β-chitin, respectively. Quantitative comparison of product formation by *Sc*LPMO10D^CD^ and two other chitin-active active LPMOs, *Sm*LPMO10A and *Cj*LPMO10A^CD^ (see below for more details), in reactions with the two chitin types showed that *Sc*LPMO10D^CD^ stands out in showing particularly low activity on α-chitin relative to the activity on β-chitin (Supplementary Fig. S4).

### Comparison of three phylogenetically distinct chitin-oxidizing LPMO10s

Figures 2 and 4 show that there is considerable sequence divergence among chitinolytic LPMOs. We set out to compare *Sc*LPMO10D^CD^ (subclade A2) with two previously characterized chitin-active LPMO10s that are phylogenetically distant from *Sc*LPMO10D^CD^, namely *Sm*LPMO10A (subclade D) and *Cj*LPMO10A^CD^ (no subclade; Fig. 2). While *Sm*LPMO10A is a single domain LPMO, *Cj*LPMO10A is naturally appended to two CBMs (AA10-CBM5-CBM73)^44^, but in this study only the catalytic domain was used for comparative purposes.

**Figure 5:**
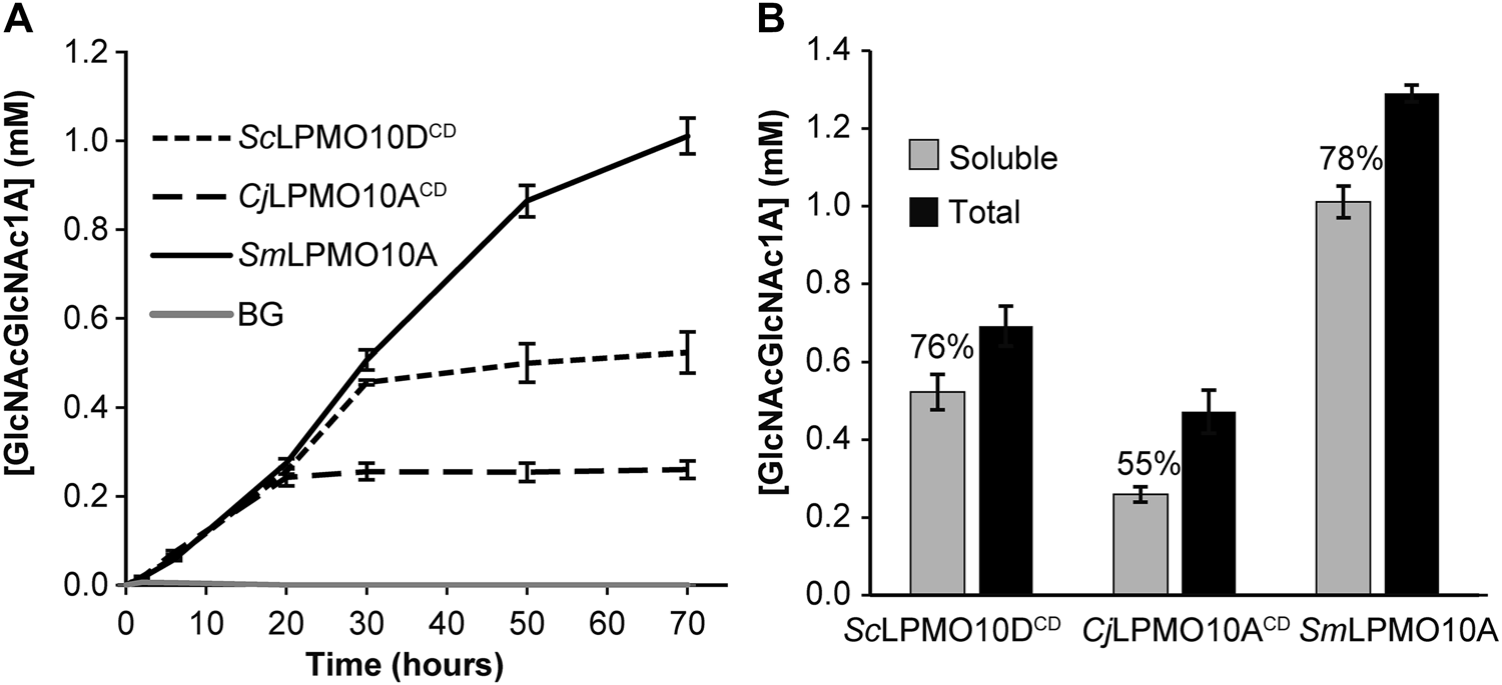
Time course of β-chitin degradation. Reactions with 1 µM LPMO** (***Sc*LPMO10D^CD^, *Cj*LPMO10A^CD^ or *Sm*LPMO10A) were incubated with 10 g/L β-chitin at 40 °C and pH 6.0 in the presence of 1 mM ascorbic acid (reducing agent) for 70 h. At various time points samples were taken and the reactions were stopped by vacuum filtering, after which the soluble oxidized products were converted to oxidized dimers via treatment with chitobiase (*Sm*CHB) prior to analysis. Panel B shows the solubilized oxidized products versus the total amount of oxidized sites in the reaction mixtures after 70 h of incubation. The total amounts of oxidized sites were determined by incubating the LPMO-treated sample at 98 °C for 10 min followed by an overnight incubation with a chitinase cocktail (2 µM *Sm*Chi18A, 2.5 µM *Sm*Chi18C, and 1 µM *Sm*CHB) at 37 °C, after which oxidized chitobiose was analyzed. The degree of solubilization in percentage is shown in the figure and the total amount of oxidized sites correspond to 100%. All reactions were performed in triplicates. The error bars show ± S.D. (n = 3).

Figure 5A shows time courses for β-chitin degradation using the three enzymes. While *Sm*LPMO10A was active for the whole period (70 h) of the measurement, product formation by the two truncated LPMOs, *Sc*LPMO10D^CD^ and *Cj*LPMO10A^CD^, ceased after 30 h and 20 h of incubation, respectively. The fraction of soluble oxidized products, relative to the total amount of oxidized products, at the end of the reaction varied from 55%, for the least stable LPMO, *Cj*LPMO10A^CD^, to 75% and 78% for the other two (Fig. 5B).

**Figure 6:**
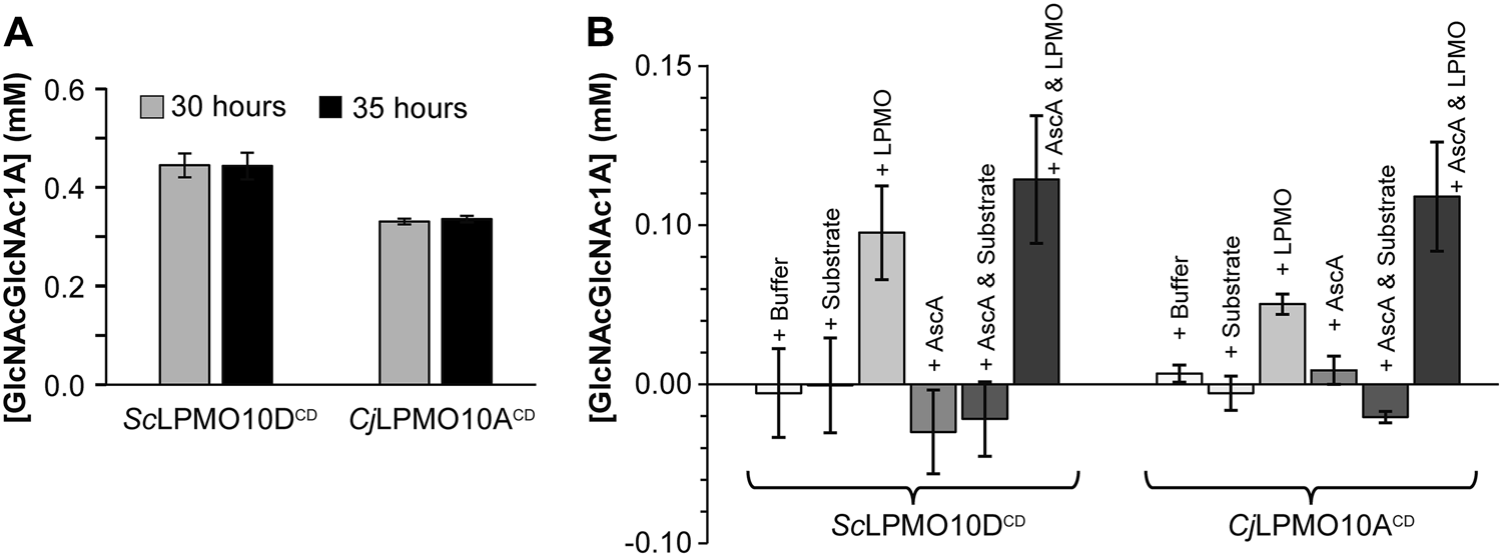
Probing the cause of inactivation of *Sc*LPMO10D^CD^ and *Cj*LPMO10A^CD^. In panel A, *Sc*LPMO10D^CD^ and *Cj*LPMO10A^CD^ were incubated at 1 μM with 10 g/L β-chitin at 40 °C and pH 6.0 in the presence of 1 mM ascorbic acid. The oxidized dimer was quantified after degradation of the solubilized oxidized products by *Sm*CHB. (**A**) Demonstrates that for both enzymes, product formation had stopped prior to 30 h of incubation, as no increase in product yield was detected between 30 and 35 h. (**B**) Shows formation of new products upon addition of fresh components to the reactions from (**A**). This was done by splitting the reaction mixtures into six equal portions, to which either buffer, substrate, LPMO, reductant, substrate + reductant, or LPMO + reductant were added, followed by incubation in standard conditions. After 13 h, all reactions were stopped by filtration before treating samples with *Sm*CHB to convert all soluble oxidized products into dimers (GlcNAcGlcNAc1A). The error bars show ± S.D. (n = 3).

It is well known that, under certain conditions, LPMOs suffer from autocatalytic oxidative damage leading to inactivation^17,46,49,50^. To assess whether the plateau in product formation in the reactions with *Sc*LPMO10D^CD^ and *Cj*LPMO10A^CD^ was caused by enzyme inactivation, or reductant or substrate depletion, an experiment was set up in which fresh reactants were added to the reactions at a point where the product formation had stopped (Fig. 6A). The data showed that only addition of fresh LPMO, alone or in combination with fresh reductant, led to recovered product formation for both enzymes (Fig. 6B), indicating that the stagnation visible in the progress curves of Fig. 5A is primarily due to inactivation of the LPMO.

The detrimental effect of removing the CBMs on the stability of *Cj*LPMO10A under turnover conditions has been correlated to the low affinity of the catalytic domain for chitin, which is weak in comparison to CBMs and natural single domain LPMOs such as *Sm*LPMO10A^44,45^. *Sc*LPMO10D does not have a CBM and binding to both chitin and cellulose has been shown for a C-terminally polyHis-tagged variant of this protein^33^. Comparative binding studies of the three LPMOs with β-chitin (Supplementary Fig. S5) showed that *Sc*LPMO10D^CD^ binds with weak affinity to β-chitin, similar to *Cj*LPMO10A^CD^, which may explain the lower stability visible in Fig. 5. Only *Sm*LPMO10A displayed a clear binding curve over time and this enzyme showed superior performance in the experiments depicted in Fig. 5.

To assess whether stability differences (Fig. 5A) could also relate to differences in folding stability, the apparent melting temperature (T_m_) of *Sc*LPMO10D^CD^ was determined by monitoring the effect of temperature on binding of a fluorescent dye (SYPRO orange). The obtained melting curve (see Supplementary Fig. S6) shows that copper-loaded *Sc*LPMO10D^CD^ has an apparent T_m_ of 63 °C which is lower compared to the previously determined apparent melting temperatures of 71.2 °C for *Sm*LPMO10A^47^ and 70.2 °C for *Cj*LPMO10A^CD^^51^, but well above the temperature used in the experiments (40 °C). Also, we confirmed the stabilizing effect of copper as shown by a ca. 10 °C drop in T_m_ for the *apo* enzyme (Supplementary Fig. S6).

### Apparent H_2_O_2_-production, -consumption and redox potential

**Figure 7:**
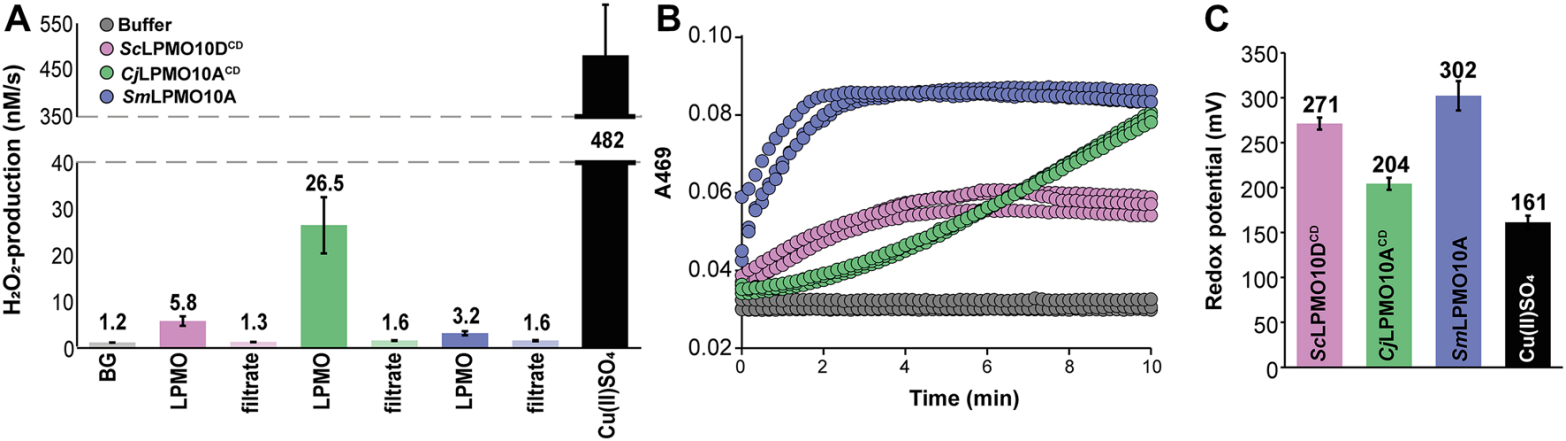
Apparent H_2_O_2_-production, -consumption, and redox potential. (**A**) Shows apparent H_2_O_2_ production by 2 µM *Sc*LPMO10D^CD^, *Cj*LPMO10A^CD^ or *Sm*LPMO10A in 50 mM sodium phosphate buffer, pH 6.0, supplied with 1 mM ascorbic acid, 5 U/ml HRP, 100 µM Amplex Red, and 1% (v/v) DMSO. Excess copper control reactions (labelled “filtrate”) were set up using protein-free samples, obtained by ultrafiltration of the protein preparations. These samples contained the same amount of free copper as the LPMO preparation used in the experiment. There are two additional control reactions: “BG”, for background, representing a reaction without enzyme, and “Cu(II)SO_4_”, a reaction in which enzyme is replaced by 2 µM CuSO_4_. (**B**) Shows the apparent peroxidase activity of the three LPMOs measured by the method described by Breslmayr et al.^54^. The reactions contained 1 µM LPMO, 1 mM 2,6-DMP, and 100 µM H_2_O_2_ in 20 mM PIPES buffer, pH 6.0. The panel shows traces for two independent reactions per enzyme. (**C**) Shows the obtained redox potentials for the LPMO-Cu^2+^/LPMO-Cu^+^ couples, determined by monitoring a reaction between 150 µM reduced *N,N,N′,N′-*tetramethyl-1,4-phenylenediamine (TMP_red_) and 35 µM (oxidized) LPMO-Cu^2+^ under anaerobic conditions. As a control, the redox potential of copper was acquired through the same method. The error bars show ± S.D. (n = 3).

To further assess differences between the three enzymes we looked at the initial oxidase rate, i.e., the rate of H_2_O_2_ generation in the absence of substrate, by using the Amplex red and horseradish peroxidase (HRP) assay as previously described^52,53^. The results showed that all three enzyme preparations were free of excess (i.e., non-LPMO bound) copper as ultrafiltrates of the enzyme preparations showed H_2_O_2_ production rates similar to the enzyme free reaction (Fig. 7A). Interestingly, *Cj*LPMO10A^CD^ which seemed the most unstable enzyme in the previous experiments (Fig. 5A), showed the highest apparent H_2_O_2_-production rate (26.5 ± 6.3 nM/s). *Sc*LPMO10D^CD^ and *Sm*LPMO10A showed significantly slower rates, amounting to 5.8 ± 1.1 nM/s and 3.2 ± 0.5 nM/s, respectively.

The peroxidase rate of the three LPMOs was measured using the assay described by Breslmayr et al.^54^. In this assay, the LPMO uses H_2_O_2_ to oxidize 2,6-dimethoxyphenol (2,6-DMP) to eventually form coerulignone, which can be detected spectrophotometrically at 469 nm. In this experiment, *Sm*LPMO10A showed the fastest consumption rate followed by *Sc*LPMO10D^CD^ and *Cj*LPMO10A^CD^ (Fig. 7B). It is worth noting that, among the three LPMOs, there is an inverse correlation between the H_2_O_2_ production rates (Fig. 7A) and the initial peroxidase rates (Fig. 7B). It is remarkable that the variation in LPMO reactivity is so different for O_2_ versus H_2_O_2_. Such a difference could be explained by the fact that catalytic rate in the peroxidase assay is limited by reduction of the LPMO by 2,6-DMP, which acts as both a reductant and a substrate^54^. In this latter case, differences in enzyme redox potentials may provide a unifying explanation: a lower redox potential is likely to lead to faster oxidation by O_2_ and slower reduction by 2,6-DMP. Indeed, one expects a correlation between the redox potential of the LPMOs and the observed oxidase activities as the first step in H_2_O_2_ production, i.e., formation of O_2_^**·**–^, is endergonic.

In accordance with the high apparent oxidase activity and low peroxidase activity of *Cj*LPMO10A^CD^, the redox potential of the *Cj*LPMO10A^CD^-Cu(II)/*Cj*LPMO10A^CD^-Cu(I) redox couple was found to be 204 ± 7 mV, which is the lowest of the LPMOs investigated in this study. In comparison, the redox potential for the *Sm*LPMO10A-Cu(II)/*Sm*LPMO10A-Cu(I) and *Sc*LPMO10D^CD^-Cu(II)/*Sc*LPMO10D^CD^-Cu(I) redox couples were determined to be 302 ± 16 mV and 271 ± 7 mV, respectively (Fig. 7C). Thus, the oxidase rates, peroxidase rates and redox potentials are correlated, and the peroxidase reaction measured in the set-up proposed by Breslmayr et al*.* is likely limited by LPMO reduction.

### Electron paramagnetic resonance (EPR) spectroscopy

The immediate environment of the active site copper ion in *Sc*LPMO10D^CD^ was analyzed by EPR spectroscopy as previously described^55^ and the EPR spectrum was simulated (Supplementary Fig. S7). The estimated spin Hamiltonian parameters are summarized in Table 2 and compared to previously published data for *Sm*LPMO10A^55^, *Sc*LPMO10C^55^ and *Cj*LPMO10A^CD^^44^. The g and A^Cu^ tensors reflect the copper coordination in the LPMO active site, and of these the g_z_ and A_z_^Cu^ could be calculated with high accuracy. The g_z_ and A_z_^Cu^ tensors for *Sc*LPMO10D^CD^ (g_z_ = 2.275; A_z_^Cu^ = 139 × 10^–4^ cm^−1^) are different compared to chitin oxidizing LPMO10s in subclade D, such as *Sm*LPMO10A, which yield values that fall between typical type 1 and type 2 copper enzymes^43,55^. With a higher A_z_^Cu^ value, the EPR spectrum of *Sc*LPMO10D^CD^ falls in between *Sm*LPMO10A (A_z_^Cu^ = 116 × 10^–4^ cm^−1^) and *Cj*LPMO10A^CD^ (A_z_^Cu^ = 154 × 10^–4^ cm^−1^). Notably, the A_z_^Cu^ of *Cj*LPMO10A^CD^resembles that of cellulose oxidizing LPMOs, such as *Sc*LPMO10C^55^, which yield EPR signals typical for type 2 copper centers^7,26,43^, based on the Peisach-Blumberg classification of type 1 and type 2 copper enzymes^56^.

**Table 2:** Comparison of LPMO Spin hamiltonian parameters.

Parameter	*Sc*LPMO10D (subclade A2)	*Cj*LPMO10A^a^ (no subclade)	*Sc*LPMO10C^b^ (subclade A1)	*Sm*LPMO10A^b^ (subclade D)	Cu(II)^a^
*g*_x_	2.021	2.036	2.015	2.039	2.059
*g*_y_	2.106	2.091	2.102	2.116	2.059
*g*_z_	2.275	2.267	2.267	2.260	2.270
*A*_x_^Cu^ ^c^	28.7	31.8	11.7	42.3	12.3
*A*_y_^Cu^ ^c^	20.6	26.1	17.0	50.3	12.3
*A*_z_^Cu^ ^c^	139	154	153	116	165

Assuming collinear *g*- and *A*^Cu^—tensors in all simulations.

^a^Data from a previous study by Forsberg et al*.*^44^.

^b^Data from a previous study by Forsberg et al*.*^56^.

^c^(10^–4^ cm^−1^).

## Discussion

In this study we have elucidated two subclades of previously uncharacterized enzymes in the AA10 phylogenetic tree that group with a subclade of strictly C1-oxidizing cellulose-active LPMOs of actinobacterial origin (subclade A1 in Fig. 2). The enzymes in subclades A2 and A3 do not only have another substrate specificity (only shown for A2) but are also different in that most of them are predicted to be cell wall anchored with (subclade A2) or without (subclade A3) crosslinking to the peptidoglycan matrix (Figs. 1, 2 and Supplementary Fig. S1).

Sequence and structural alignments (Fig. 4) show that the enzymes in subclade A3 (to which *Ma*LPMO10A belongs) have unusual active sites. For example, the so-called gatekeeper position is occupied by an aspartic acid (Asp194), whereas only Glu and Gln occur in this position in other LPMO10s (Glu203 in *Sc*LPMO10D). It is also worth noting that the A3 subclade has members in which the copper-binding histidines are replaced by glutamines (Supplementary Fig. S3). Another noteworthy observation is that *Ma*LPMO10A^CD^ and other members of subclade A3 contain a high number of arginines. The catalytic domain of *Ma*LPMO10A^CD^ (residue 27–204) contains twenty-four arginines, compared to, e.g., eight in *Sc*LPMO10D^CD^ and six in *Sm*LPMO10A. Looking closer at the position of the arginines it seems that the majority are located on the surface (Supplementary Fig. S8) and the estimated net charge of *Ma*LPMO10A^CD^ (at pH 7.4) is predicted to be + 5.03. As a comparison the predicted net charges of other LPMO10s are − 0.95 for *Sc*LPMO10D^CD^, − 3.8 for *Cj*LPMO10A^CD^, − 0.94 for *Sm*LPMO10A, − 2.24 for *Pa*LPMO10A^CD^ (CbpD^57^), − 4.93 for *Tm*LPMO10A (Tma12), − 2.94 for *Ma*LPMO10B^CD^, − 16.93 for *Sc*LPMO10B; and − 11.98 for *Sc*LPMO10C^CD^. Arginines are known to play important roles in binding negatively charged substances such as nucleic acids, cofactors, and effectors of protein active sites and for that reason some Arg-rich proteins have various interesting roles in biology, related to phenomena as diverse as gene expression, membrane-penetrating activity and pathogenesis-related defense^58^. Studies of bacterial expansins have shown huge variations in protein pI and revealed that basic expansins bind to plant cell walls via electrostatic interactions with negatively charged polysaccharides (such as pectin and some hemicelluloses), while acidic expansins are repelled^59^. It is conceivable that the high positive charge on the surface of *Ma*LPMO10A contributes to substrate binding. Another possible biological function of the positively charged residues could be to provide binding sites for protein–protein complexation between *Ma*LPMO10A and unknown partners.

*Sc*LPMO10D^CD^, our representative from subclade A2, oxidizes both α- and β-chitin with an unusually strong preference for the latter substrate (Supplementary Fig. S4. This experimental result shows that subclade A of LPMO10s contains both cellulose-active (subclade A1) and chitin-active (subclade A2) LPMOs. The crystal structure of *Sc*LPMO10D^CD^ shows that this enzyme has a “hybrid-type” of substrate binding surface similar to what is observed for *Cj*LPMO10A (Fig. 4). While residues in the second copper coordination sphere are similar to those in cellulose-active LPMOs in subclade A1, i.e., Glu203 and Arg198 in *Sc*LPMO10D (Fig. 4D), two residues which have been shown to be important for chitin activity, i.e., a pair of polar amino acids (Asn76 and Thr131 in *Sc*LPMO10D; Fig. 4D) are conserved in subclade A2, including Tma12. In the phylogenetically more distant chitin-oxidizing enzymes, such as *Sm*LPMO10A and *Cj*LPMO10A (Fig. 4B,C) and the other chitin-active LPMOs in subclades C and D shown in Fig. 4A, these residues are Gln and Thr. *Ma*LPMO10A (subclade A3) also has a polar residue pair in this position (Asp66 and Thr122; Fig. 4E), which may indicate chitin-oxidizing activity, but this remains speculative, considering the several atypical characteristics of this enzyme (discussed above) and because a conserved aromatic residue (Tyr or Trp) known to be important for binding to crystalline chitin in *Sm*LPMO10A^45^ is replaced by an Arg in *Ma*LPMO10A (Arg63; Supplementary Fig. S8) and other subclade A3 sequences.

One final interesting feature of the crystal structure of *Sc*LPMO10D^CD^ is the presence of a second arginine (Arg79), in addition to Arg198, that interacts with the catalytically crucial Glu203 (Fig. 3B). This arginine is also found in *Ma*LPMO10A and compared to subclade A2 where only 26% of the sequences possess an arginine at this position, it is fully conserved in the Arg-rich subclade A3. One would expect that the presence of a seemingly strong salt bridge involving Glu203 would affect the catalytic properties of this residue, and it would therefore be interesting to assess this in future studies. So far, we have no basis for speculating about the impact of Arg79 on LPMO reactivity.

Table 3 summarizes key properties of the three LPMO catalytic domains that are compared in this study. Comparison of the chitin-degrading abilities of *Sc*LPMO10D^CD^ (subclade A2), *Sm*LPMO10A (subclade D) and *Cj*LPMO10A^CD^ (no subclade, Fig. 2), showed mainly variation in enzyme stability. For comparative purposes we used catalytic domains only, meaning that both *Sc*LPMO1D^CD^ and *Cj*LPMO10A^CD^ lacked their linkers, CWSS or CBMs. The results show that only the naturally single module LPMO, i.e., *Sm*LPMO10A, binds well to the chitin substrate in the conditions used (Supplementary Fig. S5). Reduced LPMOs that are not bound to their substrate are more likely to take part in ‘off-pathway’ events, meaning that if a reduced LPMO reacts with H_2_O_2_ in the absence of substrate, or if the binding is weak or unprecise, for instance as a result of a CBM truncation, the reaction may lead to oxidation of the active site and inactivation of the enzyme^17,50^. It is thus not surprising that the two weak-binding LPMOs are less stable under turnover conditions.

**Table 3:** Overview of the properties of three phylogenetically distinct chitin-oxidizing LPMOs.

Enzyme	*Sc*LPMO10D^CD^	*Cj*LPMO10A^CD^	*Sm*LPMO10A	Figures
Subclade	A2	*non*	D	2
Substrate binding^a^	~ 20%	~ 20%	~ 60%	S5
T_m(app)_	63 °C	70.2 °C^b^	71.2 °C^c^	S6
Oxidase activity	5.8 nM/s	26.5 nM/s	3.2 nM/s	7A
H_2_O_2_ consumption^d^	++	+	+++	7B
Redox potential	271 mV	204 mV	302 mV	7C
Operational stability^e^	++	+	+++	5A

^a^Measured as protein adsorbed to the substrate after 2 h incubation (see Figure S5).

^b^Data from a previous study by Madland et al.^51^; pH = 7.0

^c^Data from a previous study by Jensen et al.^47^; pH = 6.0

^d^Initial activity in the 2,6-DMP assay; shown as highest (+++) to lowest (+).

^e^Stability under turnover conditions; shown as highest (+++) to lowest (+).

Another factor determining LPMO performance may be the ability of the LPMO-reductant system to generate the H_2_O_2_ co-substrate. Production of H_2_O_2_ was measured using the Amplex Red/HRP method in the absence of substrate and showed that *Cj*LPMO10A^CD^ produces some fourfold and eightfold more H_2_O_2_ compared to *Sc*LPMO10D^CD^ and *Sm*LPMO10A, respectively (Fig. 7A). In accordance with a previous observation for an LPMO11^60^, the three enzymes showed a correlation between low redox potential and high apparent oxidase activity, with *Cj*LPMO10A^CD^ having the lowest redox potential (204 ± 7 mV) and the highest apparent H_2_O_2_ production rate (26.5 ± 6.3 nM/s), followed by *Sc*LPMO10D^CD^ (redox potential: 271 ± 7 mV; H_2_O_2_ production rate 5.8 ± 1.1 nM/s) and *Sm*LPMO10A (redox potential: 302 ± 16 mV; H_2_O_2_ production rate 3.2 ± 0.5 nM/s). Of note, substrate-binding inhibits LPMO oxidase activity^49,61^. Based on these observations it is not surprising that *Cj*LPMO10A^CD^ and *Sc*LPMO10D^CD^ inactivated more rapidly than *Sm*LPMO10A, since the former two enzymes bind the substrate weakly and produce considerable amounts of H_2_O_2_, a combination that makes the enzymes prone to autocatalytic inactivation. *Sm*LPMO10A, on the other hand, shows the strongest binding and lowest H_2_O_2_ production, leading to stable progress curves.

From EPR data, redox potentials, peroxidase data and oxidase data, it is clear that the reactivities of the three LPMOs differ (see Table 3). These differences in enzyme behavior are accompanied by variation in structural features of the copper coordination sphere. The extra arginine in *Sc*LPMO10D provides one example, but cannot really be linked to functional variation, since both *Cj*LPMO10A^CD^, with higher oxidase activity, and *Sm*LPMO10A, with lower oxidase activity, lack this arginine. Further studies are needed to specifically link these structural variations to the observed functional differences.

The closest characterized homologues of *Sc*LPMO10D^CD^ are Tma12 (57.4% sequence identity), an LPMO from a fern with reported insecticidal properties^38^, and CbpD (57.3% sequence identity) from *Pseudomonas aeruginosa*, a chitin-oxidizing virulence factor that promotes survival of the bacterium in human blood^57^. Intriguingly, chitin-active LPMOs are remarkably abundant, occurring in a multitude of bacteria, some of which do not seem capable of, or at least do not regularly engage in, chitin degradation. Based on the above considerations, it may very well be that observed chitin-activity for LPMOs such as *Sc*LPMO10D and CbpD does not reflect the true biological function of these enzymes and that hitherto unknown, possibly chitin-like, LPMO substrates exist. In this respect, it is worth noting that *Sc*LPMO10D is much less active on α-chitin compared to e.g., *Sm*LPMO10A, where the latter is generally believed to play a key role in chitin degradation^62^. It is conceivable that the presence of an extra arginine in the surface-exposed catalytic center relates to substrate specificity, for example activity on negatively charged peptidoglycan.

Being Gram-positives, actinobacteria are surrounded by a thick and highly crosslinked peptidoglycan matrix which has evolved not only to protect the cells, but also to enable uptake of nutrients and secretion of metabolites such as antibiotics and proteins. The cell wall is a dynamic structure that needs to undergo changes, such as partial degradation and rebuilding, during cell differentiation and division, and it plays an import role in the interaction between actinobacterial hyphae and the environment^63^. Similar to chitin (chains of β-1,4-linked *N*-acetylglucosamine), peptidoglycan contains stretches of two amino sugars, β-1,4-linked *N*-acetylglucosamine and *N*-acetylmuramic acid (MurNAc), which are cross-linked to peptide stems. It is conceivable that the natural function of cell-wall anchored *Sc*LPMO10D relates to cell wall dynamics and turnover^33,64^. Building on the early work by Walter & Schrempf^33^, we here provide the first biochemical evidence of LPMO activity for *Sc*LPMO10D (previously called CbpC). However, we were not able to detect activity towards isolated peptidoglycan from a *Streptomyces* sp., which could be attributed to the substrate not being in its natural crystalline and insoluble context. Indeed, it is well known from research on lignocellulose-active LPMOs that certain hemicellulolytic activities are only detectable when the hemicellulose in question is adsorbed to cellulose^65^.

Streptomycetes have a complex multicellular lifestyle alternating between mycelial growth and the formation of reproductive spores, a process that involves cell wall remodeling at apical sites of the hyphae during cell elongation as well as during degradation of vegetative mycelium. Intriguingly, available transcriptomic data from Yagüe et al*.*^66^ show that *Sc*LPMO10A (SCO0481; subclade C) and *Sc*LPMO10D (SCO1734; subclade A2) are upregulated whereas *Sc*LPMO10E (SCO2833; subclade C) is downregulated when the bacteria differentiate from vegetative growth to the reproductive stage, i.e., sporulation (Supplementary Fig. S9). Interestingly, *Sc*LPMO10E has been linked to cell wall remodeling and degradation of peptidoglycan although the true substrate of this “chitin-active” LPMO remains unknown^64^. Using knock-out mutations, Zhong et al. showed that the absence of this LPMO leads to morphological changes and make the bacterium more sensitive to lysozyme^64^. Moreover, gene expression studies have shown that *S. coelicolor* only upregulates the expression of two LPMO genes when grown in chitin-rich conditions, namely *Sc*LPMO10E and *Sc*LPMO10G (SCO7225; subclade C)^30^, of which the latter displayed a five times higher increase in expression level than the former when grown in chitin-enriched soil^67^. Hence, *Sc*LPMO10G is more likely to be the main oxidizing force during chitin catabolism, while *Sc*LPMO10E seems to play a role in cell wall remodeling. Supplementary Fig. S9 shows that the down-regulation of *Sc*LPMO10E correlates with the upregulation of *Sc*LPMO10D, which we here suggest may also be involved in cellular development.

An analysis of the loci of the different LPMO genes of *S. coelicolor* showed that only *Sc*LPMO10D (SCO1734) and *Sc*LPMO10E (SCO2833) lie within the central region of the linear chromosome (SCO1440–5869;^68^). The central region encodes conserved core functions, which include cellular development^69^, while the segments flanking the central region, referred to as the left and the right arm, are suggested to encode auxiliary functions upregulated at specific conditions. The other LPMO genes are either found on the left (SCO0481, *Sc*LPMO10A; SCO0643, *Sc*LPMO10B; SCO1188, *Sc*LPMO10C) or right (SCO6345, *Sc*LPMO10F; SCO7225, *Sc*LPMO10G) arm. Thus, in line with a possible function in cellular development, one may conclude that *Sc*LPMO10D and *Sc*LPMO10E serve more critical functions, compared to the other LPMOs. It is conceivable that the relatively high number of LPMO-encoding genes in the genomes of actinobacteria (such as *S. coelicolor* and *M. aurantiaca*), compared to other prokaryotes, is related to the complex lifestyle of these organisms.

## Methods

### Bioinformatics analysis

The amino acid sequences of *Sc*LPMO10D, *Ma*LPMO10A and the six other *S. coelicolor* LPMO10s were analyzed using CAZy (http://www.cazy.org) and InterPro (http://www.ebi.ac.uk/interpro) for information on domains and functional sites as well as for identifying potential motifs for sortase-mediated cell wall-anchoring. Prediction of transmembrane helices was carried out using the TMHMM server v. 2.0 (http://www.cbs.dtu.dk/services/TMHMM).

To construct a phylogenetic tree, 150 AA10 sequences (catalytic domains only) were aligned using the MUSCLE online tool^70^ provided by EMNL-EBI. To construct the pool of sequences containing representatives from all clades of AA10 LPMOs, as previously defined by Book et al.^31^, the sequences of 45 previously characterized LPMOs (Supplementary Table S1) were used and extended by using sequences of experimentally characterized LPMOs from each clade as queries for protein–protein BLAST (blastp) searches against the non-redundant NCBI database. We aimed to add approximately 20 protein sequences to fill the subclades while not exceeding a total of 150 sequences. The added blastp sequences were selected based on their sequence identity to their queries and other subclade members, mostly rejecting sequences that did not add to the diversity of the subclades (e.g., > 90% sequence id.). The resulting multiple sequence alignment was employed as input to build the phylogenetic tree, using PhyML available via the online platform NGPhylogeny.fr^71^. The final phylogenetic tree (Fig. 2) was visualized using the iTOL platform^72^. C-terminal sequences were included to the completed phylogenetic tree to illustrate the diversity of domain architecture and other unique characteristics, such as the lack of catalytic histidines or to show sequences of non-bacterial origins.

### Cloning, expression, and purification of recombinant LPMO10s

Codon-optimized genes encoding the N-terminal domains of *S. coelicolor* A3(2) *Sc*LPMO10D, including its native signal peptide (residues 1–214; UniProt ID: Q9S296; *Sc*LPMO10D^CD^ (CD for catalytic domain)), and *M. aurantiaca* ATCC 27,029 *Ma*LPMO10A, also with its native signal peptide (residues 1–205; UniProt ID: D9TC53; *Ma*LPMO10A^CD^), were purchased from GenScript (Piscataway, NJ, USA). The genes were amplified using gene specific primers containing overhangs for cloning in the pRSET B vector (bold) and cleavage sites for *Bsm*I (underlined in the forward primers) and *Hind*III (underlined in the reverse primers), respectively, as follows; forward primer *Sc*LPMO10D^CD^ **5’-CGCAACAGGCGAATGCC**CACGGTAGCATGGGCGA-3’; reverse primer *Sc*LPMO10D^CD^ 5’-**CAGCCGGATCAAGCTT**TTAACCAAAGGTAACAT-3’; forward primer *Ma*LPMO10A^CD^ 5’- **CGCAACAGGCGAATGCC**CACGGTGCGCCGACCAG -3’; reverse primer *Ma*LPMO10A^CD^ 5’- **CAGCCGGATCAAGCTT**TTAACGAAAAATAACAT -3’. The amplified genes were inserted into pre-linearized pRSET B vector (by the two restriction endonucleases *Bsm*I and *Hind*III) containing the signal peptide of *Sm*LPMO10A (residue 1–27) using the In-Fusion® HD cloning kit (Clontech) as previously described^26^. Consequently, the native signal peptides of *Sc*LPMO10D^CD^ and *Ma*LPMO10A^CD^ is substituted for the signal peptide of *Sm*LPMO10A, which is known to result in efficient translocation of LPMOs to the periplasmic space during *Escherichia coli* expression^73^. To prepare expression strains, plasmids, whose sequence had been verified, were transformed into One Shot® BL21 Star (DE3) chemically competent *E. coli* cells (Invitrogen), according to the supplier’s protocol. Transformed cells were grown in Terrific Broth medium supplemented with 100 µg/mL ampicillin at 30 °C, using a LEX-24 Bioreactor (Harbinger Biotechnology, Canada) with compressed air for aeration and mixing. After 20 h and no induction, cells were harvested by centrifugation and periplasmic proteins were extracted using cold osmotic shock with magnesium, as previously described^74^. The resulting periplasmic fractions were sterilized by filtration through a 0.22-μm syringe filter. No soluble protein was obtained for *Ma*LPMO10A^CD^ despite several expression attempts using different types of media and different temperatures. The pH of successfully produced *Sc*LPMO10D^CD^ solution was adjusted to 9.0 by adding Tris/HCl buffer to 50 mM final concentration (buffer A), prior to protein purification. The pH-adjusted extract was loaded onto a pre-equilibrated (buffer A) 5-ml Q-Sepharose FF column (GE Healthcare) connected to an ÄKTA purifier FPLC system (GE Healthcare). Under these conditions, most native *E. coli* proteins bound to the column, whereas *Sc*LPMO10D^CD^ appeared in the flow-through. The pooled flow-through fractions were concentrated using Amicon Ultra-15 centrifugal filters with a 10 kDa molecular weight cut-off (Merck Millipore), with concomitant buffer exchange to 20 mM Tris–HCl, pH 7.5, and then loaded onto a HiLoad 16/60 Superdex 75 size-exclusion column. Fractions containing protein of high purity, analyzed by SDS-PAGE, were pooled and concentrated as described above. The protein concentration was determined by measuring absorption at 280 nm, using the theoretical extinction coefficient (34,170 M^−1^ cm^−1^)^75^ and the Beer-Lambert law.

Additional enzymes, namely the LPMOs *Cj*LPMO10A^CD^ and *Sm*LPMO10A, the chitinases *Sm*Chi18A and *Sm*Chi18C, and a GH20 chitobiase *Sm*CHB were expressed and purified as described previously^44,76^.

Before use, all LPMOs were incubated with a three-fold molar surplus of CuSO_4_ as described previously^77^, followed by desalting using PD MidiTrap G-25 columns (GE Healthcare) equilibrated with 20 mM sodium phosphate, pH 6.0. The negligible level of residual free copper in preparations of copper-saturated LPMOs was assessed by measuring the apparent H_2_O_2_ production in reactions with LPMO-free filtrates, as described below in the section “Measuring apparent H_2_O_2_ production”.

### Crystallization, diffraction data collection, structure determination, and model refinement

Crystals of the catalytic domain of *Sc*LPMO10D^CD^ (residues 34–214) were obtained with the hanging drop vapor diffusion method. Equal volumes (1 µL) of Cu(II)-saturated LPMO (in 20 mM Tris/HCl buffer, pH 8.5) and different reservoir solutions were mixed at room temperature. Crystals were obtained in 2.0 M ammonium sulfate and 0.1 M sodium cacodylate pH 6.5 at a protein concentration of 7.7 g/L. Protein crystals were soaked in a cryo-solution containing mother-liquor with 35% glucose (w/v) before they were flash frozen in liquid nitrogen. Diffraction data were collected at the ID23-1 beamline at ESRF, Grenoble, France.

Datasets were processed with XDS^78^ and scaled with POINTLESS^79^. The CCP4i2 package was used to solve the structure by molecular replacement (Phaser)^80^ and the structure was refined using REFMAC^81^. Model manipulations were carried out using Coot^82^ and molecular graphics were generated using PyMOL2 (The PyMOL Molecular Graphics System, Version 1.8, Schrödinger, LLC.).

The 3D model of *Ma*LPMO10A (UniProt ID: D9TC53, residues 27–298) without the signal peptide was built using AlphaFold 2.0.0.1 at the Saga HPC cluster (Sigma2, Norway)^48^.

### Substrate specificity of *Sc*LPMO10D^CD^

LPMO activity was primarily assessed for β-chitin (10 g/L), using 1 µM enzyme and 1 mM ascorbic acid in 50 mM sodium phosphate buffer, pH 6.0. Unless stated otherwise, all reactions were incubated in 2 mL Eppendorf tubes at 800 rpm in an Eppendorf thermomixer set to 40 °C (Eppendorf, Hamburg, Germany). Extracted and deproteinized β-chitin from squid pen (batch 20,140,101, France Chitin, Orange, France), was ball-milled and sieved to produce separate fractions of particles with 75–200 μm and 500–850 μm size. Activity was also tested towards 10 g/L shrimp shell (*Pandalus borealis*) α-chitin [purchased from Chitinor AS; Senjahopen, Norway; demineralized by hydrochloric acid treatment and subsequently deproteinized by alkaline (NaOH) treatment] and cellulosic substrates such as 5 g/L phosphoric acid swollen cellulose (PASC; prepared from Avicel PH-101 as described by Wood^83^); 10 g/L Avicel PH-101 (Sigma-Aldrich, St. Louis, MO, USA) and 1 g/L bacterial microcrystalline cellulose^84^ (kindly provided by Dr. Priit Väljamäe, University of Tartu). Furthermore, activity was tested towards chitin oligomers (DP5-6, 2 mM) purchased from Megazyme (Bray, Ireland), and peptidoglycan isolated from *Streptomyces* sp. (3 g/L) purchased from Merck (Darmstadt, Germany).

### Activity on β-chitin

Unless otherwise specified, all reactions were carried out in triplicates with 1 µM LPMO (*Sc*LPMO10D^CD^, *Sm*LPMO10A or *Cj*LPMO10A^CD^), 10 g/L β-chitin (75–200 μm particle size), and 1 mM ascorbic acid in 50 mM sodium phosphate buffer, pH 6.0. Control reactions without ascorbic acid and/or the LPMO were included in all experiments.

To quantify soluble oxidized products, aliquots were withdrawn at selected time points and the soluble fractions were immediately separated from the insoluble substrate by filtration using a 96-well filter plate (Millipore) and a Millipore vacuum manifold. By separating soluble and insoluble fractions the reactions were quenched, as the LPMOs used in this study do not oxidize soluble chito-oligosaccharides to a significant extent^44,45^. The filtrate containing solubilized oxidized chito-oligomers was subsequently mixed with an equal volume of a solution containing chitobiase from *S. marcescens* (*Sm*CHB; 1 µM final concentration) followed by an overnight incubation at 37 °C. *Sm*CHB, a GH20 β-hexosaminidase, cleaves off single GlcNAc units from chito-oligosaccharides until GlcNAc is the final product. However, if the chito-oligosaccharide is oxidized at the C1 position, as in the case for chitin-oxidizing AA10 enzymes, the final product of *Sm*CHB treatment is an oxidized dimer (chitobionic acid; GlcNAcGlcNAc1A)^77^. As a result of this treatment, all soluble oxidized products are converted to chitobionic acid, which can easily be quantified.

To determine the total amount of oxidized sites, an aliquot of the crude reaction mixture (containing both soluble and insoluble products) was diluted five times (i.e., to 2 g/L chitin), incubated at 98 °C for 10 min to terminate the LPMO reaction, and mixed with an equal volume of a chitinase cocktail (2 µM *Sm*Chi18A, 2.5 µM *Sm*Chi18C, and 1 µM *Sm*CHB, final concentrations), followed by overnight incubation at 37 °C. This treatment turned out to be sufficient to completely degrade all chitin and convert all oxidized products to chitobionic acid.

### Probing the cause of enzyme inactivation

Reactions with 10 g/L β-chitin (75–200 μm particle size) were set up using standard conditions, as described above, for all three enzymes and samples were taken and vacuum-filtered at 30 and 35 h, when product formation likely had ceased. Of note, *Sm*LPMO10A showed no signs of inactivation at these time points and was therefore excluded for the next step of the experiment. After the 35-h time-point, the remaining *Sc*LPMO10D^CD^ and *Cj*LPMO10A^CD^ reaction mixtures were divided into six identical fractions which were further supplemented with an equal volume of either (i) fresh buffer, (ii) fresh substrate (to a final concentration of 10 g/L), (iii) fresh enzyme (1 µM), (iv) fresh reductant (2 mM), (v) fresh reductant and substrate, or (vi) fresh reductant and enzyme, all in 50 mM sodium phosphate, pH 6.0. The incubation was continued overnight before terminating the reactions via filtration, followed by chitobiase treatment and quantification of chitobionic acid.

### Qualitative and quantitative analysis of oxidized chito-oligosaccharides

Product mixtures in reaction supernatants were qualitatively assessed using a matrix-assisted laser desorption/ionization time-of-flight (MALDI-ToF) UltrafleXtreme mass spectrometer (Bruker Daltonics GmbH, Bremen, Germany), equipped with a Nitrogen 337-nm laser. Reaction mixtures (1 µL) were applied to an MTP 384 ground steel target plate TF (Bruker Daltonics) together with 2 µL of 9 mg/mL of 2,5-dihydroxybenzoic acid (DHB) dissolved in 30% acetonitrile, followed by drying under a stream of air. Data collection and analysis were preformed using the Bruker FlexAnalysis software.

Quantification of oxidized chitobiose (GlcNAcGlcNAc1A) was achieved using a Dionex Ultimate 3000 UHPLC system (DionexCorp., Sunnyvale, CA, USA) equipped with a Rezex RFQ-Fast Acid H + (8%) 7.8 × 100 mm column (Phenomenex, Torrance, CA) operated at 85 °C. 8 µL samples were injected into the column and the reaction products were eluted isocratically, using 5 mM sulfuric acid as mobile phase, and detected via UV absorption at 194 nm. Data collection and analysis were performed with the Chromeleon 7.0 software. Standards were generated in-house by complete oxidation of *N*-acetyl-chitobiose (Megazyme; 95% purity) with a chitooligosaccharide oxidase from *Fusarium graminearum* (ChitO)^85^, as previously described^77^.

### Chitin binding

Binding to β-chitin in the absence of an electron donor was analyzed spectroscopically using an A_280_ method as previously described^44^. 10 g/L β-chitin (500–850 μm particle size) and 3 µM LPMO in 50 mM sodium phosphate, pH 6.0, were incubated at 40 °C and 800 rpm in an Eppendorf thermomixer (Eppendorf, Hamburg, Germany). At t = 0, 2.5, 5, 15, 30 60 and 120 min, samples were rapidly vacuum-filtered using a 96-well filter plate (0.45-μm) to separate unbound protein from protein bound to substrate. Thus, substrate binding could be monitored by measuring the A_280_, which reflects unbound protein in the reaction supernatants, at various time points.

### Apparent melting temperature (T_m_)

The apparent T_m_ of *Sc*LPMO10D^CD^, with or without the bound copper cofactor, was determined using a Protein Thermal Shift Kit (Thermo Fisher Scientific, Waltham, MA, USA), in the presence of a fluorescent dye—*SYPRO Orange*. The fluorescence of the dye is significantly increased upon binding to hydrophobic regions of the protein that become accessible as the protein unfolds^86^. A StepOnePlus Real-Time PCR machine (Thermo Fisher Scientific, Waltham, MA, USA) was used to monitor the fluorescence with Ex/Em wavelengths set to 490/530 nm. *Apo* enzyme was prepared in 50 mM sodium phosphate, pH 6.0, by preincubating *Sc*LPMO10D^CD^ in 10 mM ethylenediaminetetraacetic acid (EDTA), a metal-chelating agent, for 10 min at room temperature. Reactions were prepared in quadruplicates (*n* = 4) with 0.7 g/L copper saturated LPMO (with or without 10 mM EDTA) in 50 mM sodium phosphate, pH 6.0, and heated in the presence of the dye in a 96-well plate from 25 to 99 °C over a 50-min time period. The apparent T_m_ was derived from the first derivative of the fluorescence emission with respect to temperature (− dF/dT).

### Electron paramagnetic resonance

A 250 µL sample containing 400 µM Cu(II)-saturated *Sc*LPMO10D^CD^ in 50 mM MES, pH 6.0, was prepared and frozen in liquid nitrogen. EPR spectra were recorded using a BRUKER EleXsys 560 SuperX instrument equipped with an ER 4122 SHQE SuperX high-sensitivity cavity and a cold finger. The microwave power was set to 1 mW, the modulation amplitude was 10 G and the spectra were recorded at 77 K. The EasySpin toolbox developed for Matlab was used to simulate and fit EPR spectra^87^.

### Determination of the redox potential (E°)

The cell potentials for the redox couple Cu(II)-LPMO/Cu(I)-LPMO were determined for *Sc*LPMO10D^CD^, *Cj*LPMO10A^CD^, and *Sm*LPMO10A, as previously described^88^. For each enzyme, equal volumes (50 µL) of anaerobic solutions of reduced *N*,*N*,*N’*,*N’*-tetramethyl-1,4 phenylenediamine (TMP^red^) and Cu(II)-LPMO in 20 mM PIPES buffer (pH 6.0, T = 28 °C) were mixed to give a final concentration of 150 µM and 35 µM, respectively. The extent of the reaction was determined by detecting the absorbance of the formed TMP radical cation (TMP^ox^) at λ = 610 nm. To find the concentration of TMP^ox^, which equals the concentration of Cu(I)-LPMO, an extinction coefficient for TMP^ox^ of 14.0 mM^−1^cm^−1^ was applied^89^. From the determined concentrations of TMP^ox^ (i.e., the concentration of reduced LPMO), the equilibrium constant (*K*) was computed to yield the cell potential of the reaction between TMP^red^ and Cu(II)-LPMO. Finally, the different cell potentials for the Cu(II)-LPMO/Cu(I)-LPMO redox couples were calculated by subtracting the known cell potential for TMP^ox^/TMP^red^, 273 mV^90^, to the calculated cell potentials of the different equilibrium reactions of TMP^red^ and Cu(II)-LPMO.

### Measuring apparent H_2_O_2_ production

Production of H_2_O_2_ by the LPMO and/or through abiotic oxidation of the reductant, possibly catalyzed by free copper was analyzed according to Stepnov et al.^53^, based on the method described by Kittl et al.^52^. Reaction mixtures containing 2 µM LPMO-Cu(II), 5 U/ml horse radish peroxidase (HRP), 100 µM Amplex Red, and 1% (v/v) DMSO, were prepared in 50 mM sodium phosphate buffer, pH 6.0 and pre-incubated in a 96-well microtiter plate for 5 min at 30 °C, before initiating the reaction by adding ascorbic acid to 1 mM final concentration. Each experiment included control reactions lacking one of the reaction components, as well as H_2_O_2_ standard curves including reductant. In some of the control reactions the LPMO stock solution was substituted with an equal volume of water or with an equal volume of an LPMO-free filtrate that was prepared via ultrafiltration of the LPMO stock solution. The latter control experiments were done to establish whether the LPMO preparations contained significant amounts of free copper, as described previously^53^. For the standard curve, H_2_O_2_ dilution series were prepared in 50 mM sodium phosphate, pH 6.0 containing 5 U/ml HRP, 100 μM Amplex Red and 1 mM ascorbic acid. Hydrogen peroxide formation was monitored over time using a Varioscan LUX plate reader at 30 °C (Thermo Fisher Scientific, Waltham, MA, USA), by following resorufin absorption at 563 nm. For all reactions, the standard curve was used to convert the measured A_563_ values into micro molar concentrations of H_2_O_2_. Apparent H_2_O_2_-production rates were derived from the initial linear parts of the resorufin accumulation curves, i.e., 0.5–4 min.

### Oxidation of 2,6-DMP

*Sc*LPMO10D^CD^, *Cj*LPMO10A^CD^, and *Sm*LPMO10A were assessed for their peroxidase activity using a method described by Breslmayr et al.^54^. This assay features a spectrophotometric method with 2,6-dimethoxyphenol (2,6-DMP) as a chromogenic substrate and H_2_O_2_ as co-substrate. The LPMO carries out a peroxidase-like reaction that converts 2,6-DMP into coerulignone, the formation of which can be measured spectrophotometrically at A_469_. A solution containing 2,6-DMP and H_2_O_2_, and another solution containing the LPMO, were preincubated separately for 5 min at 30 °C in 50 mM sodium phosphate, pH 6.0. Reactions were initiated by mixing equal volumes of the two solutions, giving a final volume of 100 µL and a final concentration of 1 µM LPMO, 1 mM 2,6-DMP and 100 µM H_2_O_2_. Immediately after mixing, product formation was recorded in a Varioscan LUX plate reader (Thermo Fisher Scientific, Waltham, MA, USA), which was used to monitor the A_469_ over a period of 10 min.

## Supplementary Information

Supplementary Information.

## Data Availability

The dataset generated and analyzed during the current study is available in the Protein Data Bank (PDB ID code 7ZJB).

## Abbreviations

2,6-DMP: 2,6-Dimethoxyphenol
AscA: Ascorbic acid
AA: Auxiliary activity
CAZymes: Carbohydrate-Active enZymes
CBM: Carbohydrate-binding module
CbpC: Carbohydrate-binding protein C
CD: Catalytic domain
CHB: GH20 chitobiase
Chi18: GH18 chitinase
*Cj*: *Cellvibrio* *japonicus*
CWSS: Cell wall sorting signal
EPR: Electron paramagnetic resonance
GlcNAcGlcNAc1A: Oxidized chitobiase
GHs: Glycoside hydrolases
HRP: Horseradish peroxidase
LPMO: Lytic polysaccharide monooxygenase
*Ma*: *Micromonospora* *aurantiaca*
MALDI-ToF MS: Matrix-Assisted Laser Desorption/Ionization Time-of-Flight Mass Spectrometry
*Sm*: *Serratia* *marcescens*
*Sc*: *Streptomyces* *coelicolor* A3(2)
Tma12: *Tectaria* *macrodonta* AA10
TMP: *N,N,N’,N’*-Tetramethyl-1,4 phenylenediamine
UHPLC: Ultra-High-Performance Liquid Chromatography
