# Supplementary material for: Structural and functional characterization of the catalytic domain of a cell-wall anchored bacterial lytic polysaccharide monooxygenase from Streptomyces coelicolor
Source: Sci Rep. 2023 Apr 1;13:5345. doi: 10.1038/s41598-023-32263-7 (PMC10067821; doi:10.1038/s41598-023-32263-7)
Supplement: Supplementary file 1 — Supplementary Information. [file 41598_2023_32263_MOESM1_ESM.docx]

**Structural and functional characterization of the catalytic domain of a cell-wall anchored bacterial lytic polysaccharide monooxygenase from *Streptomyces coelicolor***

**Amanda K. Votvik^1^, Åsmund K. Røhr^1^, Bastien Bissaro^1,2^, Anton A. Stepnov^1^, Morten Sørlie^1^, Vincent G. H. Eijsink^1^ and Zarah Forsberg^1*^**

^1^ Faculty of Chemistry, Biotechnology, and Food Science, The Norwegian University of Life Sciences (NMBU), 1432 Ås, Norway

2 INRAE, Aix Marseille University, UMR1163 Biodiversité et Biotechnologie Fongiques, 13009, Marseille, France

^*^ To whom correspondence should be addressed: Zarah Forsberg ([zarah.forsberg@nmbu.no](mailto:zarah.forsberg@nmbu.no))

**SUPPORTING INFORMATION**

**Table S1.** AA10-type LPMOs that have been experimentally characterized to some extent and that have been used in the phylogenetic analysis presented in Fig. 2. The Table includes all putative AA10-type LPMOs from *S.* *coelicolor* and *M. aurantiaca,* indicated by blue and purple lettering, respectively. The four enzymes discussed in this study appear in bold face.

| **LPMO** | **Subclade^a^** | **Organism** | **Phylum** | **UniprotID** | **Domains^b^** | **PDB** | **Reference** |
| --- | --- | --- | --- | --- | --- | --- | --- |
| *Cfla*LPMO10B | A1 | *Cellulomonas flavigena* ATCC 482 | Actinobacteria | D5UGB1 | AA10-CBM2 | - | [1] |
| *Tf*LPMO10B (E8) | A1 | *Thermobifida fusca* YX | Actinobacteria | Q47PB9 | AA10-FnIII-CBM2 | - | [2] |
| *Vi*LPMO10B | A1 | *Vitiosangium* sp. GDMCC 1.1324 | Proteobacteria | A0A2T4VPM7 | AA10-FnIII-CBM2 |  | [3] |
| *Tb*LPMO10A | A1 | *Thermobispora bispora* ATCC 19993 | Actinobacteria | D6Y7U3 | AA10-CBM2 | - | [4] |
| mgLPMO10 | A1 | IMG genome ID 2199352008 | Actinobacteria | IMG/M gene ID:2200500718 | AA10-CBM2 | - | [5] |
| *Sg*LPMO10A | A1 | *Streptomyces griseus* JCM 4626 | Actinobacteria | B1VNK5 | AA10-CBM2 | - | [6] |
| *Sc*LPMO10C (CelS2) | A1 | *Streptomyces coelicolor* A3(2) | Actinobacteria | Q9RJY2 | AA10-CBM2 | 4OY7 | [7] |
| *Ma*LPMO10E | A1 | *Micromonospora aurantiaca* ATCC 27029 | Actinobacteria | D9T4V8 | AA10-CBM2 | - |  |
| *Cc*LPMO10A | A1 | *Caldibacillus cellulovorans* | - | Q9RFX5 | AA10-CBM3-GH5-CBM3 | - | [8] |
| *Tm*LPMO10A (Tma12) | A2 | *Tectaria macrodonta*(fern) | Streptophyta | W5QLL4 | AA10 | 6IF7 | [9] |
| ***Sc*LPMO10D** | **A2** | ***Streptomyces coelicolor* A3(2)** | **Actinobacteria** | **Q9S296** | **AA10-LAETG-TMH^c^** | **7ZJB** | ***This study*** |
| ***Ma*LPMO10A** | **A3** | ***Micromonospora aurantiaca* ATCC 27029** | **Actinobacteria** | **D9TC53** | **AA10-TMH^c^** | **-** |  |
| *Ma*LPMO10D | B | *Micromonospora aurantiaca* ATCC 27029 | Actinobacteria | D9T1F0 | AA10-CBM2 | - | [10] |
| *Cfla*LPMO10C | B | *Cellulomonas flavigena* ATCC 482 | Actinobacteria | D5UH31 | AA10-CBM2 | - | [1] |
| *Cfla*LPMO10A | B | *Cellulomonas flavigena* ATCC 482 | Actinobacteria | D5UGA8 | AA10-CBM2 | - | [1] |
| *Cfi*LPMO10A | B | *Cellulomonas fimi* ATCC 484 | Actinobacteria | F4H6A3 | AA10-CBM2 | - | [4] |
| *Ma*LPMO10B | B | *Micromonospora aurantiaca* ATCC 27029 | Actinobacteria | D9SZQ3 | AA10-CBM2 | 5OPF | [10] |
| *Tf*LPMO10A (E7) | B | *Thermobifida fusca* YX | Actinobacteria | Q47QG3 | AA10 | 5UIZ | [2] |
| *Sc*LPMO10B | B | *Streptomyces coelicolor* A3(2) | Actinobacteria | Q9RJC1 | AA10 | 4OY6 | [2] |
| *Kp*LPMO10A | B | *Streptomyces pratensis* ATCC 33331 / *Kitasatospora papulosa* | Actinobacteria | C9N838 | AA10 | 6NDQ | [11] |
| *Ma*LPMO10C | C | *Micromonospora aurantiaca* ATCC 27029 | Actinobacteria | D9TBD4 | AA10-CBM12 | - |  |
| *Jd*LPMO10A | C | *Jonesia denitrificans* DSM 20603 | Actinobacteria | C7R4I0 | AA10-CBM5-GH18 | 5AA7 | [12] |
| *Cfla*AA10D | C | *Cellulomonas flavigena* ATCC 482 | Actinobacteria | D5UHY1 | AA10-CBM2 | - | [1] |
| *Sg*LPMO10F | C | *Streptomyces griseus* JCM 4626 | Actinobacteria | B1VN59 | AA10 | - | [13] |
| *Sg*LPMO10B | C | *Streptomyces griseus* JCM 4626 | Actinobacteria | B1W4Z1 | AA10 | - | [13] |
| *Sc*LPMO10A | C | *Streptomyces coelicolor* A3(2) | Actinobacteria | Q9RJF2 | AA10 | - |  |
| *Sc*LPMO10G | C | *Streptomyces coelicolor* A3(2) | Actinobacteria | Q9K460 | AA10-CBM5 | - | [14] |
| *Sc*LPMO10F | C | *Streptomyces coelicolor* A3(2) | Actinobacteria | O86614 | AA10-CBM5 | - |  |
| *Sli*LPMO10E | C | *Streptomyces lividans TK24* | Actinobacteria | D6EWM4 | AA10 | 5FTZ | [15] |
| *Sc*LPMO10E | C | *Streptomyces coelicolor* A3(2) | Actinobacteria | Q9RDB8 | AA10 | - | [16] |
| *Sg*LPMO10C | C | *Streptomyces griseus* JCM 4626 | Actinobacteria | B1VW25 | AA10 | - | [13] |
| *Sg*LPMO10D | C | *Streptomyces griseus* JCM 4626 | Actinobacteria | B1VW58 | AA10 | - | [13] |
| *Bc*LPMO10A | D | *Bacillus cereus* ATCC 14579 | Firmicutes | Q81CG6 | AA10-FnIII-FnIII-CBM5 | - | [17] |
| *Bl*LPMO10A | D | *Bacillus licheniformis* ATCC 14580 | Firmicutes | Q62YN7 | AA10 | 6TWE | [18] |
| *Ba*LPMO10A | D | *Bacillus amyloliquefaciens* ATCC 23350 | Firmicutes | Q9F9Q5 | AA10 | 2YOY | [19] |
| *Ef*LPMO10A | D | *Enterococcus faecalis* V583 | Firmicutes | Q838S1 | AA10 | 4ALC | [20] |
| *Lm*LPMO10A (*Lmo*2467) | D | *Listeria monocytogenes* serotype 1/2a | Firmicutes | A0A0H3GIZ6 | AA10-FnIII-FnIII-CBM5-CBM5 | 5L2V | [21] |
| *Pl*LPMO10A | D | *Photorhabdus laumondii* subsp. *laumondii* TTO1 | Proteobacteria | Q7N4I5 | AA10 | 6T5Z | [22] |
| *Bt*LPMO10A | D | *Bacillus thuringiensis* subsp. *kurstaki* | Firmicutes | A0A0F6FRT6 | AA10 | 5WSZ | [23] |
| ***Sm*LPMO10A**  **(CBP21)** | **D** | ***Serratia marcescens* BJL200** | **Proteobacteria** | **O83009** | **AA10** | **2BEM** | **[24]** |
| *As*LPMO10A | D | *Aliivibrio salmonicida* LFI1238 | Proteobacteria | B6EQB6 | AA10-GbpA2-GbpA3-CBM73 | - | [25] |
| *Vc*LPMO10B  (GbpA) | D | *Vibrio cholerae* O1 biovar strain ATCC 39315 | Proteobacteria | Q9KLD5 | AA10-GbpA2-GbpA3-CBM73 | 2XWX | [26] |
| ***Cj*LPMO10A** | **-** | ***Cellvibrio japonicus* Ueda107** | **Proteobacteria** | **B3PJ79** | **AA10-CBM5-CBM73** | **5FJQ** | **[27]** |
| *Cj*LPMO10B | - | *Cellvibrio japonicus* Ueda107 | Proteobacteria | B3PDT6 | AA10-CBM10 | - | [28] |
| *Tt*LPMO10A | - | *Teredinibacter turnerae* ATCC 39867 | Proteobacteria | C5BKQ9 | AA10-CBM10 | 6RW7 | [29] |
| *Hc*LPMO10B (*Hc*AA10-2) | - | *Hahella chejuensis* KCTC 2396 | Proteobacteria | Q2SNS3 | AA10-IgLF-GbpA2-CBM2 | - | [30] |
| *Vi*LPMO10A | - | *Vitiosangium* sp. GDMCC 1.1324 | Proteobacteria | A0A2T4V6F4 | AA10-GbpA2-GbpA3-CBM73 | - | [3] |
| *Pa*LPMO10A (CbpD) | - | *Pseudomonas aeruginosa* UCBPP-PA14 | Proteobacteria | Q02I11 | AA10-GbpA2-CBM73 | - | [31] |
| *As*LPMO10B | - | *Aliivibrio salmonicida* LFI1238 | Proteobacteria | B6EQJ6 | AA10-GbpA2-CBM5 | - | [25] |
| *Mm*LPMO10  (*Mm*EPV fusolin) | - | *Melolontha melolontha entomopoxvirus* MMEV | Nucleocytoviricota | Q83389 | AA10-SSA | 4OW5 | [32] |
| *Wi*LPMO10  (*Wi*EPV fusolin) | - | *Wiseana* spp. entomopoxvirus | Nucleocytoviricota | A0A0J9X287 | AA10-SSA | 4YN2 | [32] |
| *Ac*LPMO10 | - | *Anomala cuprea* entomopoxvirus CV6M | Nucleocytoviricota | O70709 | AA10-SSA | 4YN1 | [32] |

^a^ According to the phylogenetic classification by Book et al. [33], with subdivision of subclade A, as outlined in the main text.

^b^ See Figure 2 for domain abbreviations.

^c^ TMH- Transmembrane helix


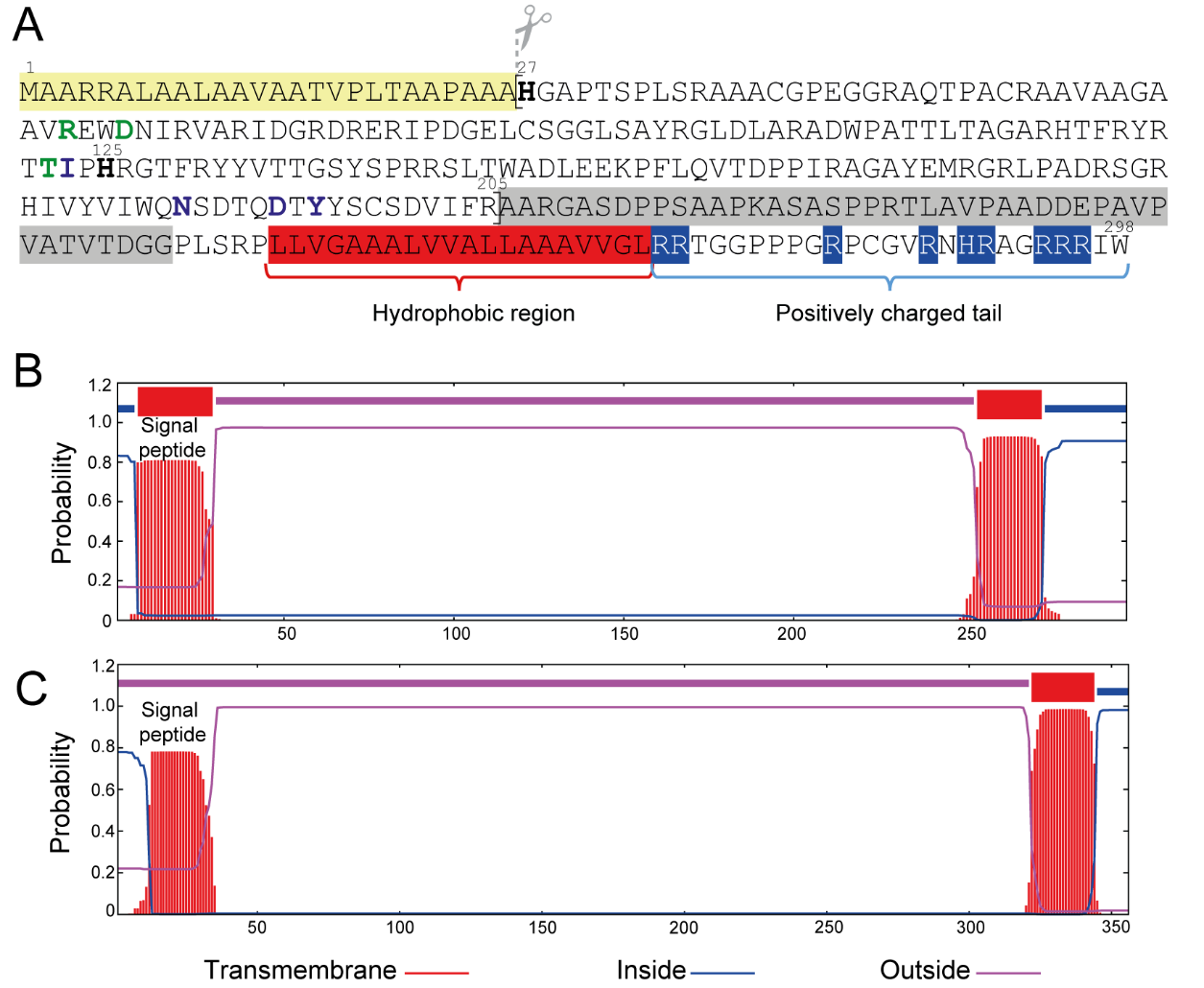


**Figure S1. Sequence analysis of *Ma*LPMO10A (A) and prediction of transmembrane helices in *Ma*LPMO10A (B) and *Sc*LPMO10D (C).** Panel A shows the primary structure of *Ma*LPMO10A, representing a group of LPMOs with a C-terminal anchoring motif but no LAETG-motif and with unusual secondary sphere active site residues (see Fig.4 and Supplementary Fig. S2 for more details). Black labelled residues show the two catalytic histidines (His27 and His125). Green residues are Asp66 and Thr122, which are typical in chitin-oxidizing LPMOs, and Arg63, which is commonly substituted by an aromatic residue (Tyr/Trp) and important for substrate binding in both cellulose- and chitin-active LPMOs. The residues printed in blue (Ile123, Asn189, Asp194 and Tyr196) are the key secondary sphere residues that are discussed in the main text. Panels B and C show results generated from prediction of transmembrane helices by the TMHMM server v. 2.0 for *Ma*LPMO10A (B) and *Sc*LPMO10D (C), respectively.


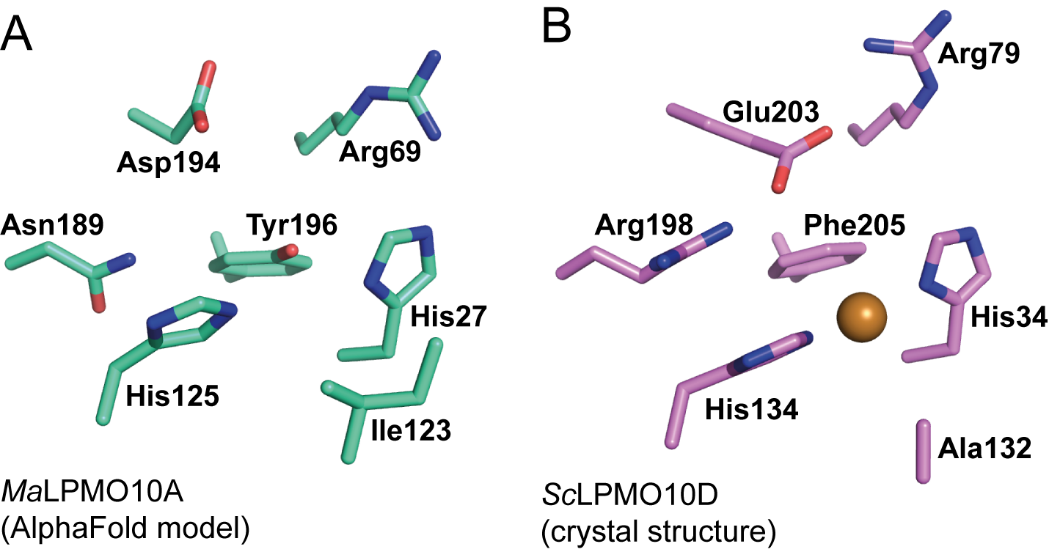


**Figure S2. Active site residues in *Ma*LPMO10A (A) and *Sc*LPMO10D (B).** The two enzymes represent two novel subclades (A2 and A3) of uncharacterized LPMOs in the AA10 phylogenetic tree (Fig. 2). A model for *Ma*LPMO10A was made using AlphaFold [34] and did not include metal ions. In the crystal structure of *Sc*LPMO10D, the copper atom is shown as an orange sphere. The so-called ‘gatekeeper’ residue, first revealed in *Sm*LPMO10A-Glu60 [35], is Glu203 in *Sc*LPMO10D and Asp194 in *Ma*LPMO10A.


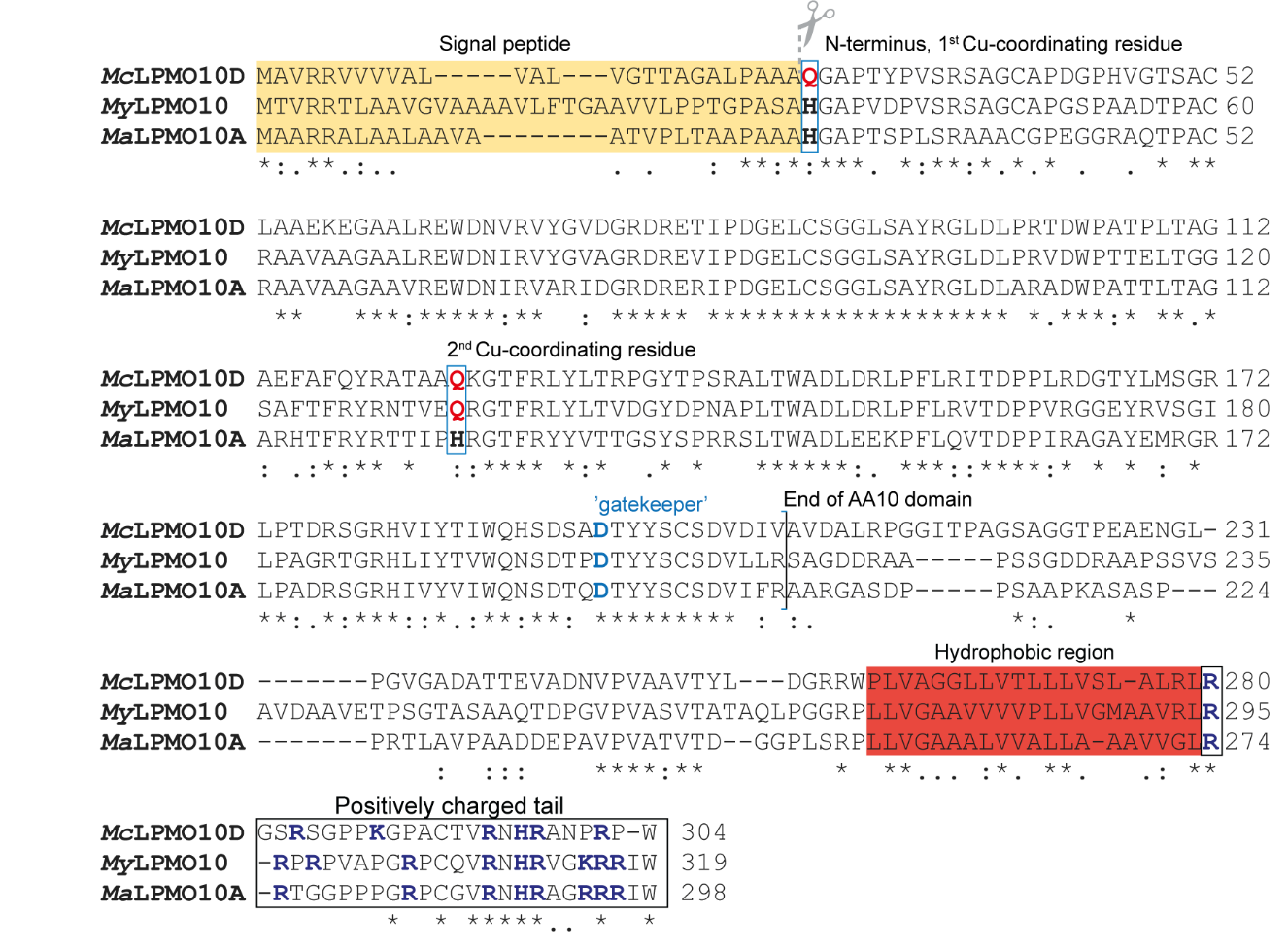


**Figure S3. Sequence alignment of *Ma*LPMO10A and two *Micromonospora* AA10 sequences that cluster in subclade A3 and lack one or two of the copper coordinating histidines**. *M. craniellae* LPMO10D (UniprotID A0A372FYB0) possesses two glutamines instead of the two catalytic histidines, whereas *M. yangpuensis* LPMO10 (UniprotID A0A1C6U851) has a glutamine instead of the second histidine. *M. aurantiaca* LPMO10A (UniprotID D9TC53) has both conserved histidines.

**
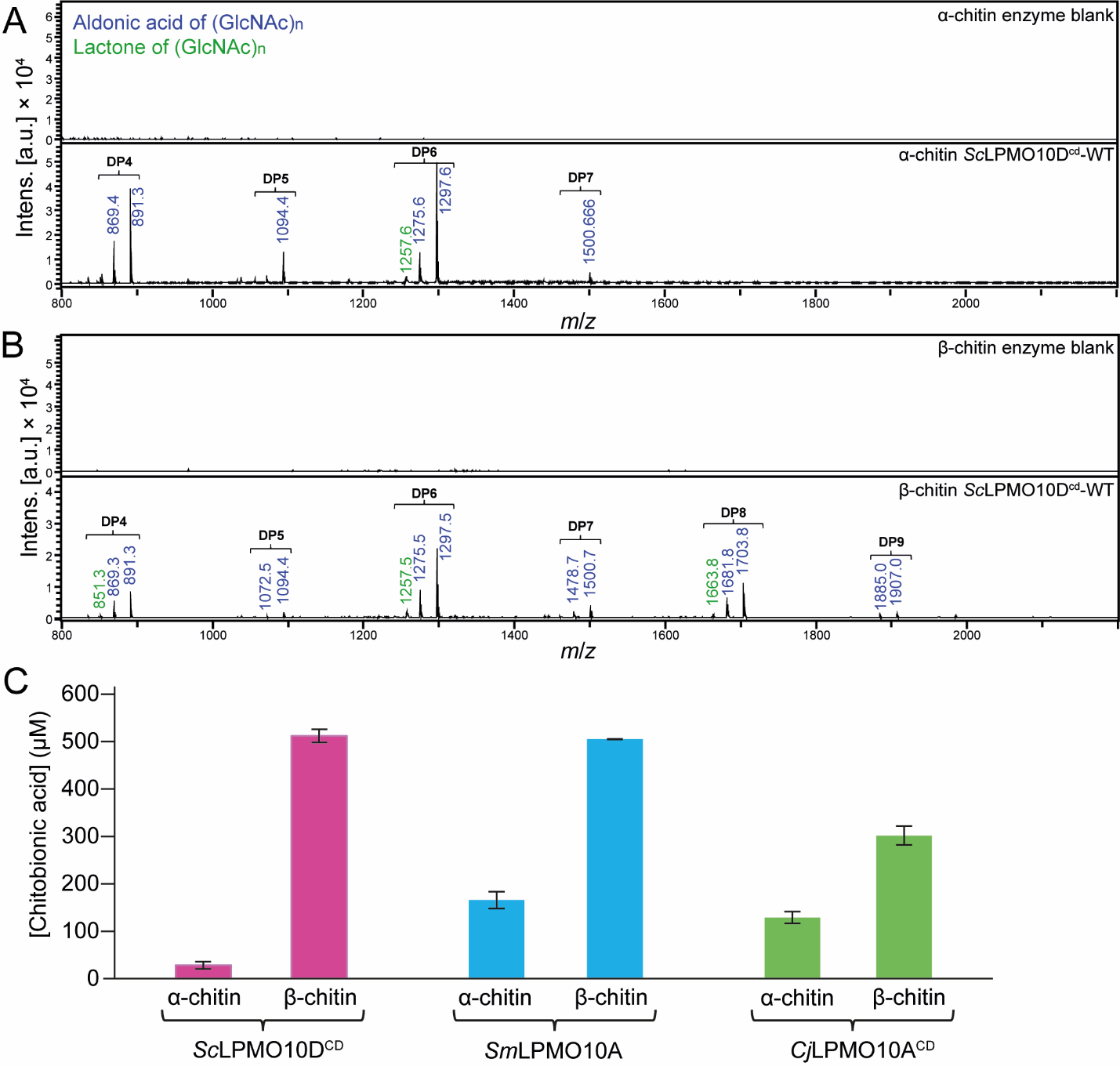
**

**Figure S4. Formation of oxidized products in reactions of *Sc*LPMO10D^CD^ with α-chitin and β-chitin.** The reactions contained 1 µM LPMO and 10 g/L chitin in 50 mM sodium phosphate pH 6.0 and were fueled by 1 mM ascorbic acid. The products shown in the mass spectra range from DP4-DP10 and correspond to sodium adducts of the δ-1,5-lactones (green; only seen for the most predominant peak clusters) and sodium adducts of the aldonic acid [GlcNAc_n_GlcNAc1A + Na]^+^ and the sodium salt of the aldonic acid [GlcNAc_n_GlcNAc1A – H + 2Na]^+^. Reactions labeled “enzyme blank” are reactions carried out without enzyme. Like the enzyme blanks, reactions without ascorbic acid did not lead to any product formation (result not shown). Panel C shows quantification of oxidized products generated in reactions containing 10 g/L shrimp-shell α-chitin or squid pen β-chitin and 1 µM LPMO and 1 mM ascorbic acid after 25 h of incubation in 50 mM sodium phosphate buffer (pH 6.0), at 40 °C and 800 rpm. The reactions were stopped by vacuum filtering and the soluble oxidized products were converted to oxidized dimers by treatment with chitobiase prior to analysis. The error bars show ± S.D. (n = 3).


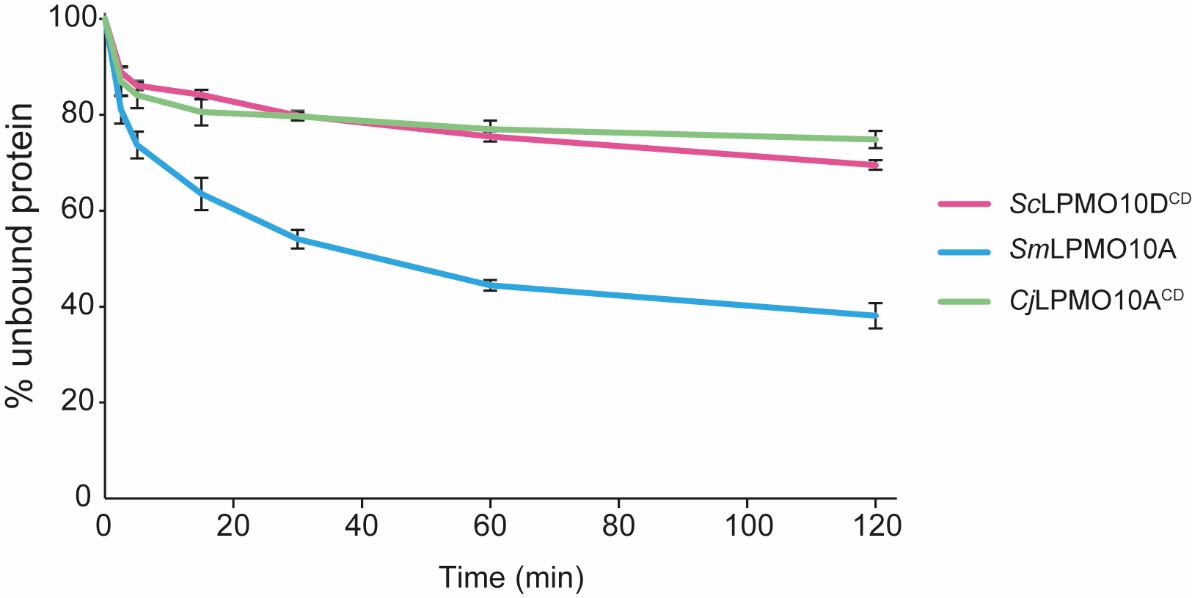


**Figure S5. Binding of LPMOs to β-chitin.** The figure shows binding as a function of time for *Sc*LPMO10D^CD^ (pink curve), *Sm*LPMO10A (blue curve) and *Cj*LPMO10A^CD^ (green curve) to 10 g/L β-chitin**.** The percentage of free LPMO was determined by measuring the reduction in protein concentration (A_280_) over time, in absence of an electron donor. The experiment was carried out at 40 °C in 50 mM sodium phosphate buffer pH 6.0 in an Eppendorf thermomixer set to 800 rpm. The error bars show ± S.D. (n = 3).

**
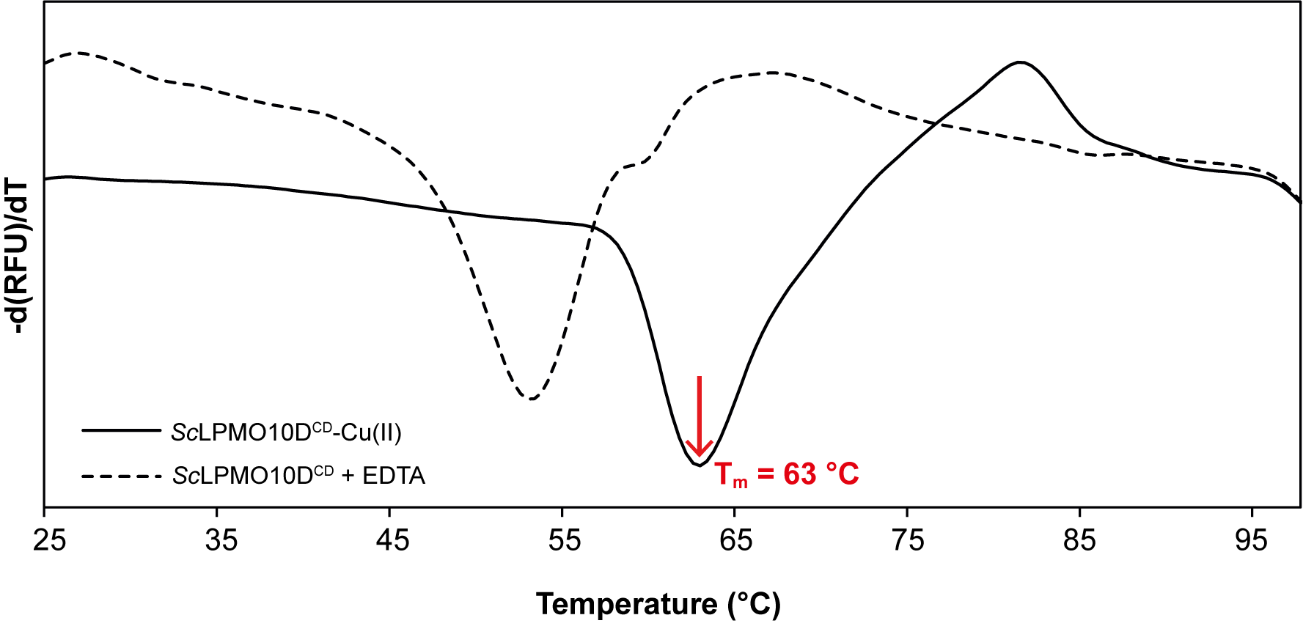
**

**Figure S6. Melting curves for *Sc*LPMO10D^CD^.** The plot displays the apparent melting temperatures (*Tm*) of *Sc*LPMO10D^CD^, either in the presence of the copper cofactor (**⎯**) or in its absence (**···**). The derivative of the fluorescence signal (-dRFU/dT) is plotted as a function of the temperature [36]. The reactions contained 0.1 g/L LPMO and were heated from 25 °C to 99 °C, at a rate of 1.0 °C /min, in the presence of SYPRO orange, a fluorescent dye. The scan was performed four times and the figure shows typical scans. The apparent *Tm* of copper-saturated *Sc*LPMO10D^CD^, indicated by the red arrow, was determined at 63 °C with a standard deviation below ± 0.1 °C. RFU – relative fluorescence units.

***
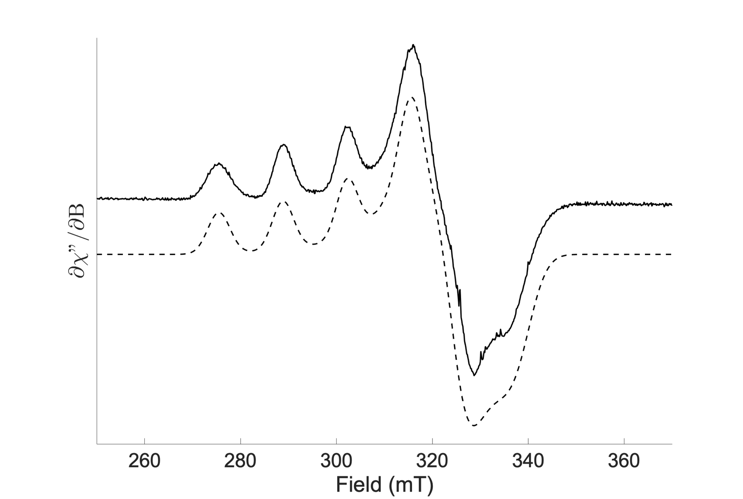
***

**Figure S7. Electron paramagnetic resonance spectroscopy of Cu(II)-saturated *Sc*LPMO10D^CD^.** X-band EPR spectrum (―) with simulation (---) for Cu(II)-saturated *Sc*LPMO10D^CD^ in 50 mM MES buffer pH 6.0. The EPR spectrum was recorded at 77 K using a microwave power of 1 mW.


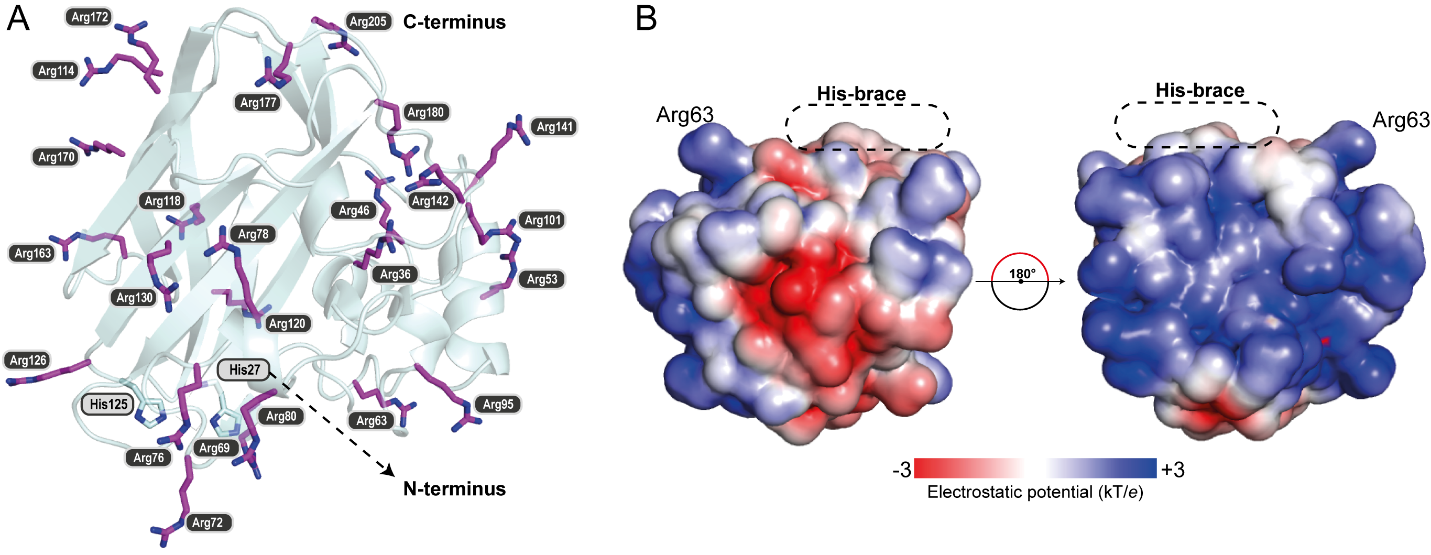


**Figure S8.** **Position of the 24 arginine residues in *Ma*LPMO10A^cd^ (A) and electrostatic potential mapped on the protein surface (B)**. Positive potential is shown in blue and negative potential in red, for a range of -3 to +3 kT/*e*. The proteins in panel B are rotated 180 degrees on the vertical axis with respect to panel A. The figures were made using PyMOL and the APBS tool was used to map the electrostatic potential.


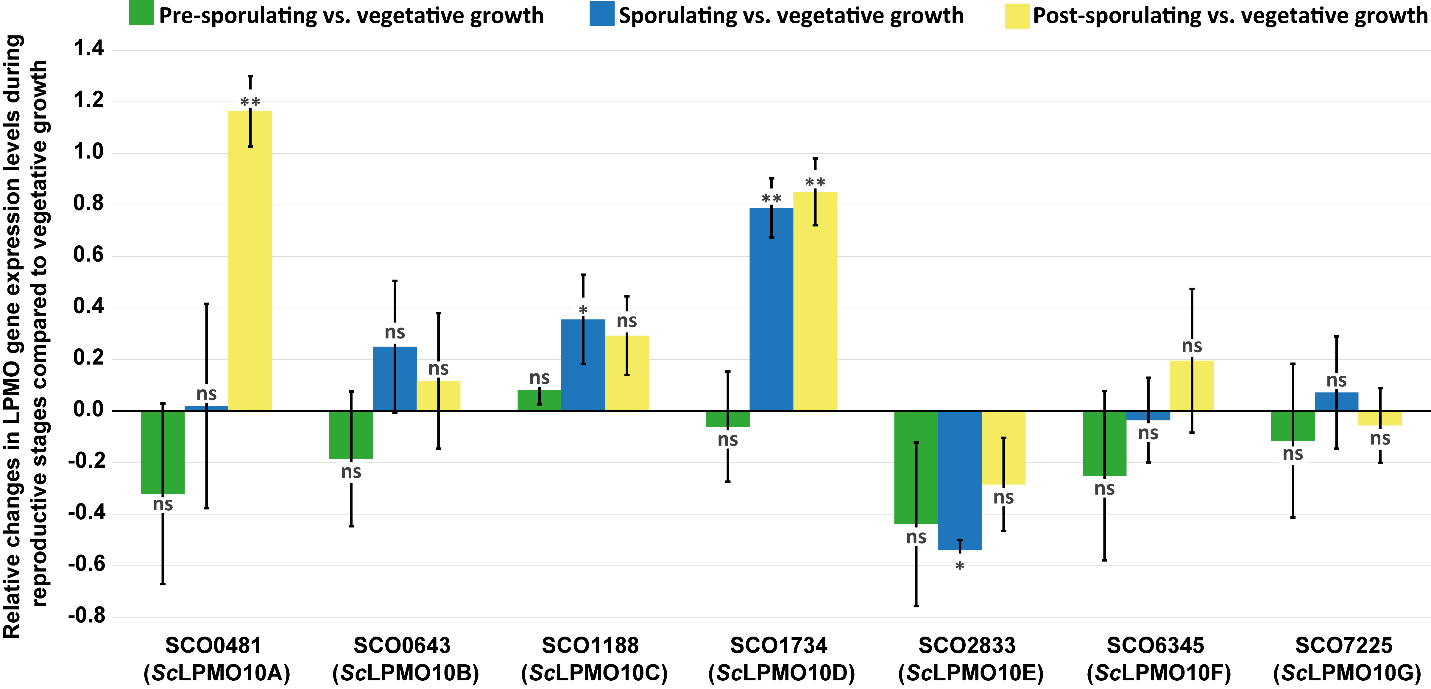


**Figure S9**. **Differences in LPMO expression observed by analyzing the transcriptomes of vegetative and reproductive (sporulating) *S. coelicolor***. The values were collected from supplementary data published by Yagüe *et al* [37] in 2013 and show that *Sc*LPMO10A (predicted chitin-active) and *Sc*LPMO10D are significantly (**; p-value ≤ 0.01) upregulated in the sporulating (only *Sc*LPMO10D) and post-sporulating stages, compared to during vegetative growth. The data also show that *Sc*LPMO10E is downregulated, while *Sc*LPMO10C is somewhat upregulated in the sporulating stage (*; p-value ≤ 0.05). Variations in the expression levels for the other stages and the other LPMOs were not significant (ns; p-value > 0.05). Relative changes in LPMO gene expression were calculated by subtracting the log2 mRNA abundance value obtained at vegetative stage from log2 mRNA abundance values obtained at reproductive stages.

**REFERENCES**

1. Li, J. *et al.* Four cellulose-active lytic polysaccharide monooxygenases from *Cellulomonas* species. *Biotechnol. Biofuels* **14**, 29; 10.1186/s13068-020-01860-3 (2021).

2. Forsberg, Z. *et al.* Structural and functional characterization of a conserved pair of bacterial cellulose-oxidizing lytic polysaccharide monooxygenases. *Proc. Natl. Acad. Sci. U. S. A.* **111**, 8446-8451; 10.1073/pnas.1402771111 (2014).

3. Zhou, X. *et al.* A myxobacterial LPMO10 has oxidizing cellulose activity for promoting biomass enzymatic saccharification of agricultural crop straws. *Bioresource Technol* **318**, 124217; 10.1016/j.biortech.2020.124217 (2020).

4. Crouch, L. I., Labourel, A., Walton, P. H., Davies, G. J. & Gilbert, H. J. The contribution of non-catalytic carbohydrate binding modules to the activity of lytic polysaccharide monooxygenases. *J. Biol. Chem.* **291**, 7439-7449; 10.1074/jbc.M115.702365 (2016).

5. Tuveng, T. R. *et al.* A thermostable bacterial lytic polysaccharide monooxygenase with high operational stability in a wide temperature range. *Biotechnol. Biofuels* **13**, 194; 10.1186/s13068-020-01834-5 (2020).

6. Sato, K. *et al.* Functional analysis of a novel lytic polysaccharide monooxygenase from *Streptomyces griseus* on cellulose and chitin. *Int. J. Biol. Macromol.* **164**, 2085-2091; 10.1016/j.ijbiomac.2020.08.015 (2020).

7. Forsberg, Z. *et al.* Cleavage of cellulose by a CBM33 protein. *Protein Sci.* **20**, 1479-1483; 10.1002/pro.689 (2011).

8. Sunna, A., Gibbs, M. D., Chin, C. W., Nelson, P. J. & Bergquist, P. L. A gene encoding a novel multidomain beta-1,4-mannanase from *Caldibacillus cellulovorans* and action of the recombinant enzyme on kraft pulp. *Appl. Environ. Microb.* **66**, 664-670; 10.1128/AEM.66.2.664-670.2000 (2000).

9. Yadav, S. K., Archana, Singh, R., Singh, P. K. & Vasudev, P. G. Insecticidal fern protein Tma12 is possibly a lytic polysaccharide monooxygenase. *Planta* **249**, 1987-1996; 10.1007/s00425-019-03135-0 (2019).

10. Forsberg, Z. *et al.* Structural determinants of bacterial lytic polysaccharide monooxygenase functionality. *J. Biol. Chem.* **293**, 1397-1412; 10.1074/jbc.M117.817130 (2018).

11. Corrêa, T. L. R. *et al.* An actinobacteria lytic polysaccharide monooxygenase acts on both cellulose and xylan to boost biomass saccharification. *Biotechnol. Biofuels* **12**, 117; 10.1186/s13068-019-1449-0 (2019).

12. Mekasha, S. *et al.* Structural and functional characterization of a small chitin-active lytic polysaccharide monooxygenase domain of a multi-modular chitinase from *Jonesia denitrificans*. *FEBS Lett.* **590**, 34-42; 10.1002/1873-3468.12025 (2016).

13. Nakagawa, Y. S. *et al.* Analysis of four chitin-active lytic polysaccharide monooxygenases from *Streptomyces griseus* reveals functional variation. *J. Agr. Food. Chem.* **68**, 13641-13650; 10.1021/acs.jafc.0c05319 (2020).

14. Li, F., Zhao, H., Liu, Y., Zhang, J. & Yu, H. Chitin biodegradation by lytic polysaccharide monooxygenases from *Streptomyces coelicolor in vitro* and *in vivo*. *Int. J. Mol. Sci.* **24**, 275; 10.3390/ijms24010275 (2023).

15. Chaplin, A. K. *et al.* Heterogeneity in the histidine-brace copper coordination sphere in auxiliary activity family 10 (AA10) lytic polysaccharide monooxygenases. *J. Biol. Chem.* **291**, 12838-12850; 10.1074/jbc.M116.722447 (2016).

16. Zhong, X. B., Zhang, L., van Wezel, G. P., Vijgenboom, E. & Claessen, D. Role for a lytic polysaccharide monooxygenase in cell wall remodeling in *Streptomyces coelicolor*. *mBio.* **13**, e0045622; 10.1128/mbio.00456-22 (2022).

17. Mutahir, Z. *et al.* Characterization and synergistic action of a tetra-modular lytic polysaccharide monooxygenase from *Bacillus cereus*. *FEBS Lett.* **592**, 2562-2571; 10.1002/1873-3468.13189 (2018).

18. Courtade, G. *et al.* Mechanistic basis of substrate-O_2_ coupling within a chitin-active lytic polysaccharide monooxygenase: An integrated NMR/EPR study. *Proc. Natl. Acad. Sci. U. S. A.* **117**, 19178-19189; 10.1073/pnas.2004277117 (2020).

19. Hemsworth, G. R. *et al.* The copper active site of CBM33 polysaccharide oxygenases. *J. Am. Chem. Soc.* **135**, 6069-6077; 10.1021/ja402106e (2013).

20. Vaaje-Kolstad, G. *et al.* Characterization of the chitinolytic machinery of *Enterococcus faecalis* V583 and high-resolution structure of its oxidative CBM33 enzyme. *J. Mol. Biol.* **416**, 239-254; 10.1016/j.jmb.2011.12.033 (2012).

21. Paspaliari, D. K., Loose, J. S. M., Larsen, M. H. & Vaaje-Kolstad, G. *Listeria monocytogenes* has a functional chitinolytic system and an active lytic polysaccharide monooxygenase. *FEBS J.* **282**, 921-936; 10.1111/febs.13191 (2015).

22. Munzone, A. *et al.* Characterization of a bacterial copper-dependent lytic polysaccharide monooxygenase with an unusual second coordination sphere. *FEBS J.* **287**, 3298-3314; 10.1111/febs.15203 (2020).

23. Zhang, H. Y., Zhao, Y., Cao, H. L., Mou, G. Q. & Yin, H. Expression and characterization of a lytic polysaccharide monooxygenase from *Bacillus thuringiensis*. *Int. J. Biol. Macromol.* **79**, 72-75; 10.1016/j.ijbiomac.2015.04.054 (2015).

24. Vaaje-Kolstad, G. *et al.* An oxidative enzyme boosting the enzymatic conversion of recalcitrant polysaccharides. *Science* **330**, 219-222; 10.1126/science.1192231 (2010).

25. Skåne, A. *et al.* The fish pathogen *Aliivibrio salmonicida* LFI1238 can degrade and metabolize chitin despite gene disruption in the chitinolytic pathway. *Appl. Environ. Microb.* **87**, e0052921; 10.1128/AEM.00529-21 (2021).

26. Wong, E. *et al.* The *Vibrio cholerae* colonization factor GbpA possesses a modular structure that governs binding to different host surfaces. *PLoS Pathog.* **8**, e1002373; 10.1371/journal.ppat.1002373 (2012).

27. Forsberg, Z. *et al.* Structural and functional analysis of a lytic polysaccharide monooxygenase important for efficient utilization of chitin in *Cellvibrio japonicus*. *J. Biol. Chem.* **291**, 7300-7312; 10.1074/jbc.M115.700161 (2016).

28. Gardner, J. G. *et al.* Systems biology defines the biological significance of redox-active proteins during cellulose degradation in an aerobic bacterium. *Mol. Microbiol.* **94**, 1121-1133; 10.1111/Mmi.12821 (2014).

29. Fowler, C. A. *et al.* Discovery, activity and characterisation of an AA10 lytic polysaccharide oxygenase from the shipworm symbiont *Teredinibacter turnerae*. *Biotechnol. Biofuels* **12**, 232; 10.1186/s13068-019-1573-x (2019).

30. Ghatge, S. S. *et al.* Multifunctional cellulolytic auxiliary activity protein *Hc*AA10-2 from *Hahella chejuensis* enhances enzymatic hydrolysis of crystalline cellulose. *Appl. Microbiol. Biotechnol.* **99**, 3041-3055; 10.1007/s00253-014-6116-6 (2015).

31. Askarian, F. *et al.* The lytic polysaccharide monooxygenase CbpD promotes *Pseudomonas aeruginosa* virulence in systemic infection. *Nat. Commun.* **12**, 1230; 10.1038/s41467-021-21473-0 (2021).

32. Chiu, E. *et al.* Structural basis for the enhancement of virulence by viral spindles and their in vivo crystallization. *Proc. Natl. Acad. Sci. U. S. A.* **112**, 3973-3978; 10.1073/pnas.1418798112 (2015).

33. Book, A. J. *et al.* Evolution of substrate specificity in bacterial AA10 lytic polysaccharide monooxygenases. *Biotechnol. Biofuels* **7**, 109; 10.1186/1754-6834-7-109 (2014).

34. Jumper, J. *et al.* Highly accurate protein structure prediction with AlphaFold. *Nature* **596**, 583-589; 10.1038/s41586-021-03819-2 (2021).

35. Bissaro, B., Isaksen, I., Vaaje-Kolstad, G., Eijsink, V. G. H. & Røhr, Å. K. How a lytic polysaccharide monooxygenase binds crystalline chitin. *Biochemistry-Us* **57**, 1893-1906; 10.1021/acs.biochem.8b00138 (2018).

36. Huynh, K. & Partch, C. L. Analysis of protein stability and ligand interactions by thermal shift assay. *Curr. Protoc. Protein Sci.* **79**, 28.29.21-28.29.14; 10.1002/0471140864.ps2809s79 (2015).

37. Yagüe, P. *et al.* Transcriptomic analysis of *Streptomyces coelicolor* differentiation in solid sporulating cultures: first compartmentalized and second multinucleated mycelia have different and distinctive transcriptomes. *Plos One* **8**, e60665; 10.1371/journal.pone.0060665 (2013).
